# Predominance of *Cand*. Patescibacteria in Groundwater Is Caused by Their Preferential Mobilization From Soils and Flourishing Under Oligotrophic Conditions

**DOI:** 10.3389/fmicb.2019.01407

**Published:** 2019-06-20

**Authors:** Martina Herrmann, Carl-Eric Wegner, Martin Taubert, Patricia Geesink, Katharina Lehmann, Lijuan Yan, Robert Lehmann, Kai Uwe Totsche, Kirsten Küsel

**Affiliations:** ^1^Aquatic Geomicrobiology, Institute of Biodiversity, Friedrich Schiller University Jena, Jena, Germany; ^2^German Centre for Integrative Biodiversity Research (iDiv) Halle-Jena-Leipzig, Leipzig, Germany; ^3^Hydrogeology, Institute of Geosciences, Friedrich Schiller University Jena, Jena, Germany

**Keywords:** shallow subsurface, ultra-small bacteria, oligotrophy, community assembly, co-occurrence, *Cand*. Patescibacteria, *Cand*. Paceibacteria

## Abstract

Despite the widely observed predominance of *Cand*. Patescibacteria in subsurface communities, their input source and ecophysiology are poorly understood. Here we study mechanisms of the formation of a groundwater microbiome and the subsequent differentiation of *Cand*. Patescibacteria. In the Hainich Critical Zone Exploratory, Germany, we trace the input of microorganisms from forested soils of preferential recharge areas through fractured aquifers along a 5.4 km hillslope well transect. *Cand*. Patescibacteria were preferentially mobilized from soils and constituted 66% of species-level OTUs shared between seepage and shallow groundwater. These OTUs, mostly related to *Cand*. Kaiserbacteraceae, *Cand*. Nomurabacteraceae, and unclassified UBA9983 at the family level, represented a relative abundance of 71.4% of the *Cand*. Patescibacteria community at the shallowest groundwater well, and still 44.4% at the end of the transect. Several *Cand*. Patescibacteria subclass-level groups exhibited preferences for different conditions in the two aquifer assemblages investigated: *Cand*. Kaiserbacteraceae surprisingly showed positive correlations with oxygen concentrations, while *Cand*. Nomurabacteraceae were negatively correlated. Co-occurrence network analysis revealed a central role of *Cand*. Patescibacteria in the groundwater microbial communities and pointed to potential associations with specific organisms, including abundant autotrophic taxa involved in nitrogen, sulfur and iron cycling. Strong associations among *Cand*. Patescibacteria themselves further suggested that for many groups within this phylum, distribution was mainly driven by conditions commonly supporting a fermentative life style without direct dependence on specific hosts. We propose that import from soil, and community differentiation driven by hydrochemical conditions, including the availability of organic resources and potential hosts, determine the success of *Cand*. Patescibacteria in groundwater environments.

## Introduction

In recent years, the bacterial tree of life underwent a tremendous expansion through the discovery of the immense microbial diversity within the ‘candidate phyla radiation’ (CPR) (Hug et al., 2016). Recent phylogenetic and taxonomic analyses suggested the reclassification of the CPR as a single phylum, *Cand*. Patescibacteria (Parks et al., 2018), with 14 classes known so far. The majority of these taxa were predicted based on metagenomic analysis of habitats difficult to access, such as groundwater, deep sea sediments, permafrost, and the continental deep subsurface (Brown et al., 2015; Luef et al., 2015; Frey et al., 2016; Hubalek et al., 2016; Léon-Zayas et al., 2017). In fact, groundwater environments have turned out to contain a particularly high abundance of *Cand*. Patescibacteria, up to 38% of the total microbiomes (Bruno et al., 2017; Kumar et al., 2017; Schwab et al., 2017). As available information about these organisms is derived almost exclusively from (meta)genomic analyses, research needs to be aimed at elucidating their origin and ecophysiology to understand their success in these habitats.

Seventy-five to eighty-four percent of the cells in groundwater environments were previously found to be so-called low-nucleic acid (LNA) content bacteria as defined by flow cytometry, usually corresponding to cells smaller than 0.4 μm (Besmer et al., 2016; Proctor et al., 2018). Members of *Cand*. Patescibacteria in groundwater are especially abundant in the ultra-small fraction of cells (Miyoshi et al., 2005; Luef et al., 2015), i.e., cells that even pass through 0.2 μm pore size filters (Rappé et al., 2002; Wurch et al., 2016; Castelle et al., 2018). The widespread use of such filters for biomass collection likely contributed to the oversight of these microorganisms in past studies. In oligotrophic habitats like pristine groundwater, ultra-small cell size is thought to be evolutionarily advantageous, as the increased surface-to-volume ratio optimizes uptake of the sparse nutrients (Sowell et al., 2009). Lack of nutrients alone might further lead to a reduction of cell size in starving microorganisms (Hood and Macdonell, 1987; Vybiral et al., 1999; Young, 2006). Typically associated with inherently small cell sizes is a reduction in genome size by loss of expendable genes, which leads to a lower metabolic cost of reproduction (Giovannoni et al., 2014). Taken to the extreme, this can result in the loss of essential metabolic functions, which inevitably leads to dependencies on other organisms. Members of *Cand*. Patescibacteria, e.g., *Cand*. Paceibacteria or *Cand*. Microgenomatia, often show such reduced genomes of approximately 1 Mbp, and a lack of functional genes essential for amino acid or nucleotide biosynthesis (Brown et al., 2015), and hence, a host-dependent lifestyle of these organisms has been suggested (Hug et al., 2015; Castelle et al., 2018). Therefore, abundance and community structure of *Cand*. Patescibacteria in groundwater might not just be dependent on the ambient hydrochemical conditions, but also the availability of partners might shape their distribution patterns.

Despite the widely observed predominance of *Cand*. Patescibacteria in subsurface microbial communities, surprisingly little attention has been paid to the origin of these organisms and to the mechanisms by which they are introduced into groundwater and ultimately become the dominant members of the groundwater microbiome. In this work, we aimed to identify potential sources of *Cand*. Patescibacteria in groundwater, and assess key factors underlying their establishment and differentiation, focusing on groundwater hydrochemical conditions as well as potential interactions with other members of the groundwater microbial community as inferred from co-occurrence networks. We traced members of *Cand*. Patescibacteria along a 5.4 km soil and groundwater monitoring transect in the hillslope terrain of the Hainich Critical Zone Exploratory (CZE), a unique field site which allowed us to study the formation of the groundwater microbiome in the common geologic setting of thin-bedded mixed carbonate-siliciclastic bedrock (Küsel et al., 2016). We accessed soil seepage of the forested surface-recharge area, and upper slope shallow perched groundwater at 5 m depth down to downslope resources in fractured bedrock strata at about 90 m below the surface. Our results suggest the soils as the origin of *Cand*. Patescibacteria, as these organisms are readily mobilized with seepage and constitute the largest fraction of taxa shared between seepage and shallow groundwater. Within the groundwater, divergent trends in the preference for several hydrochemical parameters resulted in the differentiation of *Cand*. Patescibacteria communities across the two aquifer assemblages of our study site. Co-occurrence networks pointed to potential interactions with other bacterial groups, including autotrophs. However, the distribution patterns of various *Cand*. Patescibacteria groups appeared to be independent of specific partner organisms.

**FIGURE 1:**
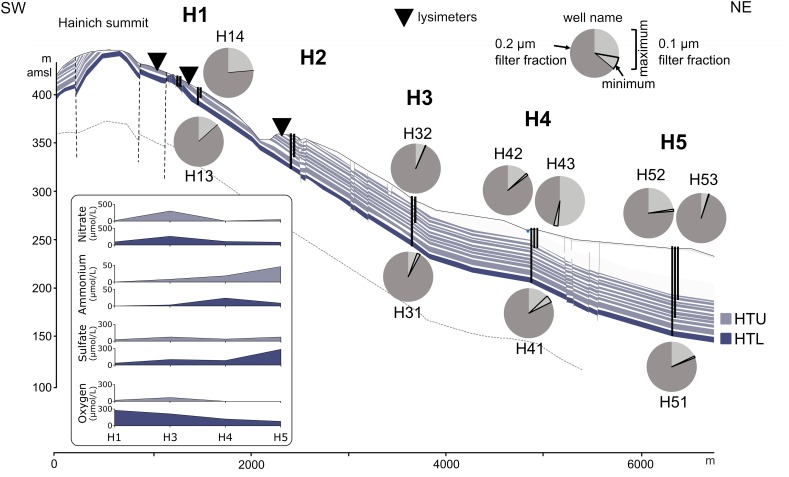
Proportions of ultra-small cells in 0.1 and 0.2 μm pore size filter fractions from groundwater samples along the eastern Hainich hillslope, aquifer configuration, and selected physicochemical parameters. Groundwater wells are shown as vertical black lines. The cross section [from Kohlhepp et al. (2017), modified] shows the karstified main aquifer [HTL; (wells H13, H31, H41, H51)] that is characterized by massive limestone beds and higher surface-connection by hilltop preferential recharge areas. The hanging thin-bedded alternating limestone-mudstone strata that host the multi-story upper aquifer assemblage (HTU; wells H14, H32, H42, H43, H52, H53) exhibit lower hydraulic conductivities due to a lesser intensity of karstification, yielding longer groundwater residence times. Faults are indicated by vertical dashed lines. Lysimeter installations used in this study are shown as inverted triangles. Quantification was based on 16S rRNA gene targeted qPCR (H13, H14: one sampling time point; other wells: two (H31) or six sampling time points). Dark gray section of the pie chart represents proportion of bacterial 16S rRNA genes associated with the 0.2 μm filter fraction. Light gray section of the pie chart represents the maximum (encircled area: minimum) proportion of bacterial 16S rRNA genes associated with the 0.1 μm filter fraction.

## Materials and Methods

### Study Site, Sampling, and Chemical Analysis

Soil materials, seepage, and groundwater were collected from the Hainich CZE located in Thuringia, Germany, which was established in the framework of the Collaborative Research Center AquaDiva (Küsel et al., 2016). The location, geological setting, construction procedures as well as materials of groundwater wells were described elsewhere in detail (Küsel et al., 2016; Kohlhepp et al., 2017; Lazar et al., 2019). Outcropping bedrocks and aquifer strata of the hillslope terrain belong to the lithostratigraphic subgroup Upper Muschelkalk of the Germanic Triassic (Kohlhepp et al., 2017). The sloping strata of thin-bedded marine limestone-mudstone alternations host a multi-story aquifer system of a local groundwater flow system. Two aquifer assemblages were sampled: the limestone-dominated, karstified lower aquifer assemblage (HTL) and the mudstone-dominated upper aquifer assemblage (HTU) (Küsel et al., 2016; Figure 1). To follow the vertical transfer of microorganisms from soils (Chromic Cambisol; Cambisol) via seepage, we utilized tension-supported lysimeters (METER Group AG, Munich, Germany) installed in duplicates in 30 cm depth at a hilltop monitoring plot representing the forested preferential surface-recharge area of the sloping strata (Kohlhepp et al., 2017) (H1L1-1, H1L1-2; H1L3-1, H1L3-2: managed forest; H2L1-1, H2L1-2: unmanaged forest). The lysimeters were composed of a stainless-steel ring (diameter: 30 cm; height: 10 cm) filled with glass beads (size/diameter: ∼2 mm) to support and hydraulically connect the overburden, undisturbed soil, along with a porous silicon carbide suction plate (SIC320; pore size of ∼20 μm; same manufacturer) at the bottom. Suction was applied via a battery powered vacuum controller (VS-twin, same manufacturer) and regulated according to the prevailing soil matrix potential, measured constantly with a tensiometer (T8, same manufacturer).

For hillslope shallow groundwaters (ten wells) and recharge areas (3 plots) six to eight sampling time points were integrated (see Supplementary Material), complemented with a one-time sampling of forest top soil (TS; 10 cm depth) in five spatial replicates (samples H1-TS1 – H1-TS5, H2a-TS1 – H2a-TS5) in vicinity to the lysimeter installations at two locations (H1L1, H2L1) in September 2016 (*n* = 10). Soil samples were obtained using a sterile spatula, transferred to sterile 50 ml tubes, frozen on dry ice, and stored at −80°C until nucleic acid extraction. Number of temporal replicates differed across groundwater wells and lysimeters because not all the sampling sites yielded enough water for analysis at all six time points. Regular sampling of the groundwater and chemical analyses were described elsewhere (Küsel et al., 2016; Kohlhepp et al., 2017). Groundwater samples for molecular analysis were obtained in September, November, and December 2015 and in June, August, and November 2016 and were collected in autoclaved 10 L FLPE (fluorinated polyethylen) containers and kept at 4°C until filtration was performed within 1 h. Groundwater samples were filtered through sterile 0.2 μm polycarbonate filters (Nuclepore, Whatman), and the filtrate was collected and subsequently filtered through sterile 0.1 μm polycarbonate filters. The filtered volumes ranged from 6 to 20 L. Lysimeter samples were obtained at eight time points between November 2016 and March 2017 and were filtered through sterile 0.2 μm pore size and subsequently through sterile 0.1 μm pore size polyethersulfone (PES) filters (Supor, Pall Corporation), with 100–400 ml collected on one filter. All filters were stored at −80°C until nucleic acid extraction was performed.

### Nanoparticle Tracking Analysis

Measurements of the size distribution and concentration of mobile particles in groundwater samples were performed by nanoparticle tracking analysis (NTA) using an NS500 instrument (NanoSight; Malvern Instruments Ltd., Worcestershire, United Kingdom), equipped with a light source (diode laser, 405 nm, power <60 mW), a high-sensitive CMOS-camera system, and video analysis software.

### DNA Extraction, Quantitative PCR, and Amplicon Sequencing

DNA was extracted from soil, groundwater, and seepage filter retentates using the PowerSoil DNA Isolation Kit (MO BIO Laboratories, CA, United States) following the manufacturer’s protocol. Abundances of bacterial 16S rRNA genes were determined by quantitative PCR (qPCR) on a Mx3000P instrument (Agilent, Böblingen, Germany) using Maxima SYBR Green Mastermix (Thermo Fisher Scientific, Germany) and the primer combinations Bac8Fmod/Bac338Rabc (Daims et al., 1999; Loy et al., 2002) following cycling conditions previously described (Herrmann et al., 2012). Amplicon sequencing of bacterial 16S rRNA genes was carried out using the primer combination Bakt_341F/Bakt_805R (Herlemann et al., 2011). Generation of barcoded amplicons and amplicon sequencing using the Illumina MiSeq platform and V3 Chemistry (Illumina) was performed by LGC Genomics (Berlin, Germany) as previously described (Kumar et al., 2017). Sequence analysis of bacterial 16S rRNA amplicons was performed using Mothur (v.1.39.1) (Schloss et al., 2009), following the Mothur MiSeq SOP (Kozich et al., 2013) along with the SILVA bacteria reference alignment v132 (Quast et al., 2013) as previously described (Kumar et al., 2017). To implement the genome-based phylogeny recently proposed by Parks et al. (2018), resulting high quality bacterial 16S rRNA sequence reads were subsequently classified against the 16S rRNA reference database of the Genome Taxonomy Database (GTDB release 03-RS86, reference file bac_ssu_r86.1_20180911, provided at the GTDB website^1^). For all analyses that used sequence information from the total community, that is, merged information from the 0.1 and 0.2 μm filter fraction, we used 16S rRNA gene qPCR data to calculate how much each filter fraction contributed to the total community for a given sample. In the next step, we multiplied relative abundances of taxonomic groups in each filter fraction with these correction factors and used the sum of the corrected relative abundances in the 0.1 and 0.2 μm filter fraction as relative abundance of a given taxonomic group within the total community.

Sequence data obtained in this study have been deposited in the European Sequence Archive (ENA; accession numbers ERS2221375-ERS2221502 in bioproject PRJEB25133).

### Estimation of Mechanisms of Community Assembly

The relative importance of deterministic selection vs. stochastic processes on bacterial community assembly was evaluated by pairwise community comparison based on the turnover in phylogenetic community composition and species composition using a null model approach according to Stegen et al. (2013, 2015). The R code for this analysis was provided by Stegen et al. (2013). This approach assumes that species of more close phylogenetic relationships share more similar ecological niches. The abundance-weighted β-mean-nearest taxa distance (βMNTD) was computed to evaluate the pairwise phylogenetic turnover between a given pair of communities with the R package picante (Kembel et al., 2010). The null distribution of βMNTD values was generated via 999 times of randomization under the null hypothesis that the bacterial communities have identical phylogenetic composition. During each randomization, the species names were moved randomly across the tips of the phylogeny and one βMNTD was calculated. β-nearest taxon index (βNTI) was calculated to represent the difference between observed βMNTD and the mean of the null distribution in standard deviation units. β-Nearest Taxon Index (βNTI) infers the relative importance of selection (βNTI > 2: variable selection; βNTI < −2: homogeneous selection) and stochasticity (| βNTI| < 2) in bacterial community assembly. To further characterize the mechanisms that underlie the stochastic processes in the bacterial community assembly, the re-scaled Raup-Crick probability index RC_bray_ (Chase et al., 2011) was calculated based on Bray-Curtis distance. The null distribution of RC_bray_ values was generated via 999 times of randomization under the null hypothesis that the bacterial communities have identical species composition. When the environmental selection is low (| βNTI| < 2), an RC_bray_ value less than −0.95 or over 0.95 indicates that homogenizing dispersal or dispersal limitation is the dominant assembly process, respectively. An RC_bray_ value between −0.95 and 0.95 suggests no dominant assembly process.

Calculations integrated data from the connected wells (H13, H31, H41 and H51 in the lower aquifer assemblage) taken in August 2016. Prior to analysis, data sets were subsampled to 7876 reads per sample, and the sequence information from the 0.1 and 0.2 μm filter fraction was merged for each groundwater well and time point. OTUs with low read numbers were retained in the data set. The phylogenetic tree was generated from the aligned sequences of the representative OTUs in Mothur based on the relaxed neighbor-joining method (Evans et al., 2006). Calculations were carried out using the R code provided by the original authors at github^2^.

### Co-occurrence Network Analysis

Network analyses were carried out using the R software framework (v. 3.4.2) (R Core Team, 2014) and the packages Matrix (v. 1.2.3)^3^, igraph (1.1.2) (Csárdi and Nepusz, 2006), and SpiecEasi (v. 0.1.2) (Kurtz et al., 2015) including respective dependencies. OTU abundance information from the 0.1 and 0.2 μm filter fractions was merged for each site and time point prior to analysis to perform network analysis on the total community. We further filtered out OTUs that were represented by less than 100 sequence reads over all datasets or that were not present in at least 30% of all datasets. This step was included to minimize the interference from OTUs in network analysis that are only present in few samples or at low abundance, and to reduce the computational load for the network construction. This decomplexed OTU table contained 854 out of the original 189600 OTUs. These 854 OTUs accounted for 68% of the total sequence reads obtained from groundwater. The decomplexed OTU table was subsequently subjected to co-occurrence network reconstruction using Meinshausen–Bühlmann neighborhood selection (Meinshausen and Bühlmann, 2006) as an inference model in SpiecEasi. The settings were as follows:

lambda.min.ratio=1e-2, nlambda=20, icov.select.params=list (rep.num=50). Edge confidence values representing edge stability and reproducibility were calculated based on random re-sampling of the data using the model selection scheme StARS (Stability Approach to Regularization Selection) (Liu et al., 2010). An igraph network object for downstream analysis was subsequently created based on afore-mentioned edge confidence values. The network was filtered for positive interactions (defined as positive model coefficients) before being analyzed for inherent network clusters using the cluster_greedy function of igraph. High confidence edges (edge confidence > 0.5) were extracted, the network re-clustered, and individual clusters were inspected by subgraphing based on cluster members. Network characteristics were assessed by analyzing edge confidence frequencies as well as degree distributions.

### Growth Experiments, Flow Cytometry, and Transmission Electron Microscopy

To test the effect of organic carbon availability on cell size, 26 heterotrophic bacterial groundwater isolates previously obtained on Reasoners2A medium (Reasoner and Geldreich, 1985) at 15°C in the dark and taxonomically characterized by 16S rRNA gene sequencing (Supplementary Table 1) were cultivated with different concentrations of organic carbon. The strains were pre-incubated for 2 days in a modified liquid R2A medium, containing 0.6 g/L K_2_HPO_4_ × 3H_2_O, 0.1 g/L MgSO_4_ × 7H_2_O, 0.6 g/L sodium pyruvate, 1.0 g/L peptone, 1.0 g/L caseinhydrolysat, 1.0 g/L yeast extract, and 1.0 g/L dextrose; corresponding to 880.0 mg/L C_org_, for 2 days. The cultures were then centrifuged, and the cell pellets were washed twice in sterile 1 M NaCl solution to remove residual medium prior to inoculation of the cultures of the main experiment. To observe the adjustment of cell size to different C_org_ concentrations, the medium described above was used (a) in its undiluted version and (b) with carbon sources diluted to 0.088 mg/L C_org_. All cultures were set up with a volume of 40 mL in 50 mL Greiner BioOne Cultivation tubes. 1 g of sterile silica beads was added to every cultivation tube in order to detach all cells from the tube walls by vortexing prior to measuring cell size distribution by flow cytometry. Incubations were carried out at 15°C in the dark under constant agitation. A control was run along with all dilution levels of C_org_. After 5 days of cultivation, cell size distributions were analyzed by flow cytometry. Of each culture, 990 μl were incubated with 10 μl of SYBR Green II (Invitrogen) for 10 min at room temperature in the dark. Analysis was performed in a CyFlow Cube 6 (Sysmex, Germany), measuring forward scatter (FSC, related to cell size) and green fluorescence emission measured at 530 ± 30 nm (FL1) using a 488 nm laser, to discriminate and enumerate bacterial cells. All cytometric analyses were evaluated on a logarithmic scale using the FCS Express 5 Flow Research Editions (DeNovo) software. Stained cells were visually distinguished from background by plotting the FL1 versus FSC signal of the negative controls. For further analyses only, events that were identified as cells were considered and the median of all FSC values in one sample was calculated within the software as a proxy of cell size within every sample. One isolate (hainich_200, *Flavobacterium* sp.) was selected for TEM analysis on a Zeiss CEM 902 A electron microscope (Carl Zeiss AG, Oberkochen, Germany). Cell material was fixed with 2.5% (v/v) glutaraldehyde in cacodylate buffer (100 mM, pH 7.4) for 2 h at room temperature. Fixed samples were subsequently washed three times with cacodylate buffer, and post-fixed with 1% osmium tetroxide in cacodylate buffer for 2 h at 20°C. Next, samples were dehydrated in an ascending ethanol series and stained with 2% (w/v) uranyl acetate in 50% (v/v) ethanol. The samples were embedded in Araldite resin (Plano, Wetzlar, Germany), ultrathin sections (70 nm thickness) were cut using an ultramicrotome Ultracut E (Reichert-Jung, Vienna, Austria), and mounted on Formvar-carbon coated 100 mesh grids (Quantifoil, Großlöbichau, Germany). Ultrathin sections were stained with lead nitrate for 10 min (Venable and Coggeshall, 1965) and examined in a Zeiss CEM 902 A electron microscope (Carl Zeiss AG, Oberkochen, Germany) and imaged using a TVIPS 1k Fast-Scan CCD-Camera (TVIPS, Munich, Germany).

### Statistical Analysis

Correlations between chemical parameters and relative abundances of OTUs as well as taxonomic groups were assessed using Spearman rank correlation coefficients (two-sided) in PAST (Hammer et al., 2001). Differences of first-degree neighbors in co-occurrence networks were determined using Mann–Whitney *U* test in PAST.

**FIGURE 2:**
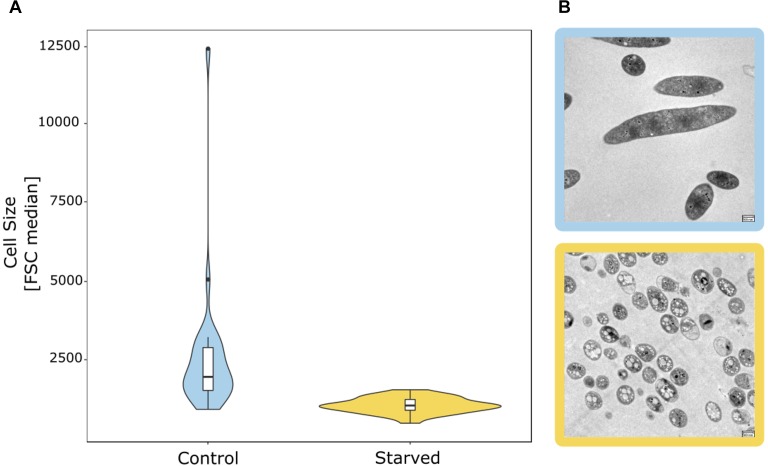
Starvation-induced reduction in the cell size of bacterial groundwater isolates. **(A)** Distribution of the median FSC signal of 26 isolates as a proxy for cell size in cultures grown in full medium with 880 mg/L C_org_ (control) vs. starved cultures with 0.088 mg/L C_org_. **(B)** Change of cell size and morphology based on TEM pictures of groundwater isolate hainich_200 (affiliated with *Flavobacterium* sp.) from full medium culture (blue frame) to starved culture (yellow frame). Scale bar: 500 nm.

## Results

Across all the groundwater wells of the Hainich CZE, organisms of the *Cand*. Patescibacteria represented the largest fraction of the groundwater microbial communities, with relative abundances ranging from 17 to 79%. *Cand*. Patescibacteria were especially enriched in the ultra-small fraction of cells: Following sequential filtration of groundwater through 0.2 and 0.1 μm pore size filters, up to 83% of the community collected on the 0.1 μm pore size filters were related to class *Cand*. Paceibacteria (groups previously referred to as *Cand*. Parcubacteria; Brown et al., 2015; Hug et al., 2016) of the phylum *Cand*. Patescibacteria (Supplementary Figure 1A). The eight most abundant taxa within the class *Cand*. Paceibacteria showed enrichment factors between 1.9- and 4.1-fold in relative abundance between the 0.2 and 0.1 μm pore size fraction (Supplementary Figure 2). Classes *Cand*. Microgenomatia and *Cand*. Saccharimonadia of the *Cand*. Patescibacteria likewise displayed a tendency to occur in the 0.1 μm filter fraction, with respective enrichment factors of 2.1 and 1.3. Conversely, members of classes ABY1 and *Cand*. Gracilibacteria, were strongly reduced in relative abundance in the ultra-small fraction, with enrichment factors of 0.7-fold to less than 0.0001-fold. To estimate how the population of a given taxonomic group was distributed across the 0.1 and 0.2 μm filter fraction, we combined qPCR-based information of bacterial abundances with sequencing data (Supplementary Figure 3). For several family- and order- level groups of *Cand*. Paceibacteria, between 15 and 30% of their total population were estimated to pass through the 0.2 μm filters.

The majority of microbial taxa with cultured representatives were predominantly found in the 0.2 μm filter fraction (Supplementary Figures 2, 3), with the exception of spore forming *Firmicutes* and *Spirochaetota*, whose spiral shape at a diameter lower than 0.2 μm might allow them to pass through the filters. Consequently, well-studied microbial taxa such as *Proteobacteria*, *Nitrospirota*, *Planctomycetota*, *Bacteroidota*, and *Actinobacteriota* feature larger cell sizes, which agrees with previous flow-cytometry-based findings by Proctor et al. (2018). However, even members of these groups were occasionally detected in the 0.1 μm filter fraction. Using 26 heterotrophic bacterial strains of these taxa obtained from the Hainich CZE (Supplementary Table 1), we tested whether a reduction of cell size due to starvation in the oligotrophic groundwater could explain this phenomenon. In fact, 80% of these isolates showed reduced cell sizes based on the forward scatter (FSC) signal in flow cytometric analysis (Figure 2), when incubated for 5 days in conditions resembling the pristine groundwater in comparison to full medium with 880 mg L^−1^ C_org_. Of note, one *Flavobacterium* isolate (hainich_200) showed a 96% decrease of the FSC signal in the starved cultures. Transmission electron microscopy confirmed a strong reduction of cell size, from 3.2 ± 2.0 μm to 0.98 ± 0.19 μm, along with a drastic change in cell shape (Figure 2). The isolates used were affiliated with bacterial genera which we had also detected in the groundwater bacterial communities based on 16S rRNA gene targeted amplicon sequencing. Relative abundances of the respective sequence reads suggested that these genera represented 6.9% of the total groundwater bacterial community. Hence, this wide-spread tendency for a reduction of cell size under nutrient limited conditions might explain why, apart from the dominant *Cand*. Patescibacteria, also classical heterotrophs were found in the ultra-small fraction.

The relative abundances of such ultra-small cells were surprisingly high, comprising up to 54% of the groundwater bacterial populations (Figure 1 and Supplementary Figure 4), as determined by qPCR. Bacterial 16S rRNA gene abundances were 1.2 × 10^7^ – 8.6 × 10^8^ genes L^−1^ groundwater for the 0.2 μm filter fraction and 3.3 × 10^5^ – 8.1 × 10^7^ genes L^−1^ for the 0.1 μm filter fraction (Supplementary Table 2). Mean fractions of ultra-small cells in each well along the groundwater transect ranged from 2.0 to 19.7%. These estimates are rather conservative, given that some ultra-small cells will be retained on 0.2 μm filters, e. g., if they occur in aggregates or due to filter clogging at higher particle load. Similarly, ultra-small bacteria affiliated with *Cand*. Patescibacteria probably harbor only one 16S rRNA operon (Brown et al., 2015) while operon numbers may be in the range of one (*Cand*. Patescibacteria, *Thermodesulfovibrionia*, *Nitrospirota*, *Brocadiae*) or two to four (*Alpha*- and *Gammaproteobacteria*) for the bacteria commonly observed in the communities on the 0.2 μm filters [information derived from rrnDB-website^4^ (Stoddard et al., 2015)]. These differences may additionally lead to an underestimation of the fraction of ultra-small cells.

**FIGURE 3:**
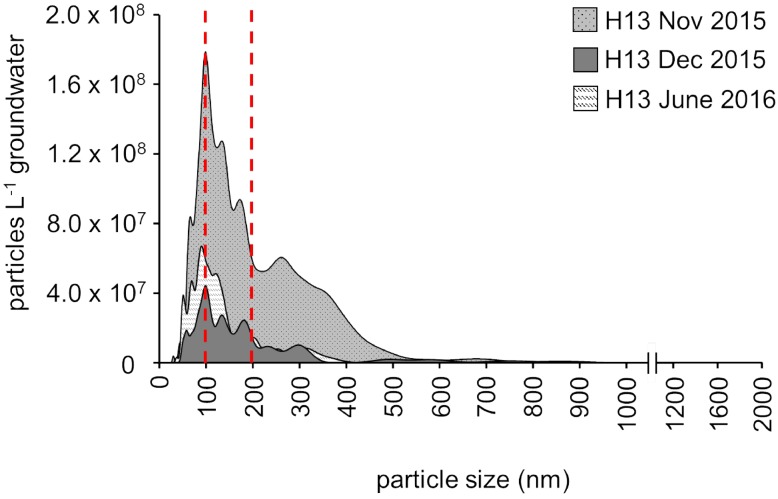
Content and size distribution of groundwater particles from the first well of the transect, H13 (5.1 m below the surface), assessed by nanoparticle tracking analysis (NTA) for three different time points (November 2015, December 2015, January 2016; one replicate per time point). The red lines indicate filter pore size commonly used for microbial biomass collection in environmental surveys (0.2 μm) and the (0.1 μm) filter pore size additionally used here.

Nanoparticle tracking analysis measurements, providing an unbiased picture of the particle load, in fact revealed that in the shallow groundwater at the hilltop position of our groundwater well transect, more than 60% of the particles were consistently smaller than 0.2 μm (Figure 3). The unique design of our monitoring wells, however, allows for the sampling of suspended particles up to five millimeters in size (Küsel et al., 2016) These particles, comprising not only organisms, but also inorganic and organic material, are mobilized by infiltrating precipitation from soils through weathered rocks and into the groundwater. With 4.0 × 10^9^ to 2.2 × 10^10^ particles per L in the well at the hilltop position, this translocation from surface to subsurface might be an important entry point for ultra-small cells of *Cand*. Patescibacteria into the groundwater. To identify the origin of *Cand*. Patescibacteria hence required us to trace back the flow of water to the soils of the preferential recharge area.

### *Cand*. Patescibacteria Are Readily Mobilized From Soils

One great strength in the design of the Hainich CZE monitoring transect is the ability to follow the formation of the groundwater microbiome. Microbial populations can be traced from their potential origin, forest soils in the preferential surface-recharge area, vertically in seepage collected at 30 cm depth, down to upper slope shallow perched groundwater and downslope groundwater in the fractured limestone-mudstone alternations.

**FIGURE 4:**
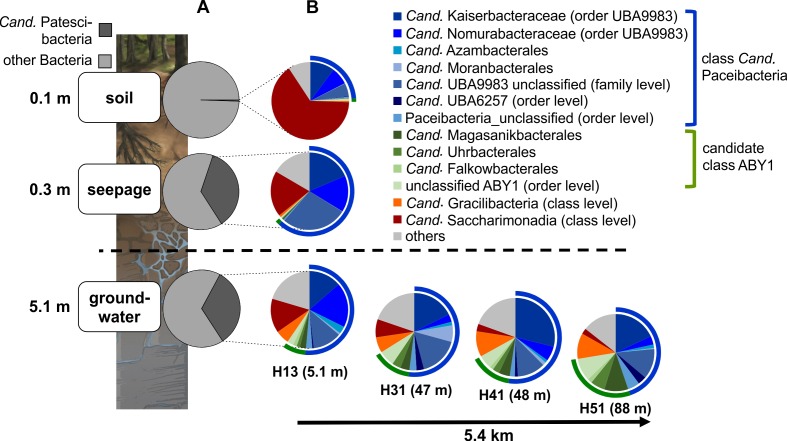
Community structure of *Cand*. Patescibacteria in soil, seepage and groundwater. **(A)** Changes in the relative fraction of *Cand*. Patescibacteria within the bacterial communities from soil via seepage to groundwater and **(B)** changes in the order-level community structure of *Cand*. Patescibacteria (family level for *Cand*. Kaiserbacteraceae, *Cand*. Nomurabacteraceae and unclassified UBA9983; class level for *Cand*. Gracilibacteria and *Cand*. Saccharimonadia) from soil to seepage and groundwater and along the groundwater observation transect (lower aquifer assemblage). Data are based on 16S rRNA gene-targeted Illumina MiSeq amplicon sequencing and represent mean values of 10 spatial replicates for soil, mean values from five lysimeters with each one sampling time point except for H1L1-2 (*n* = 6) (total *n* = 11), and one (H13), two (H31) or six different sampling time points for groundwater (total *n* = 15). Seepage and groundwater samples integrate data from the 0.1 and 0.2 μm filter fractions.

**FIGURE 5:**
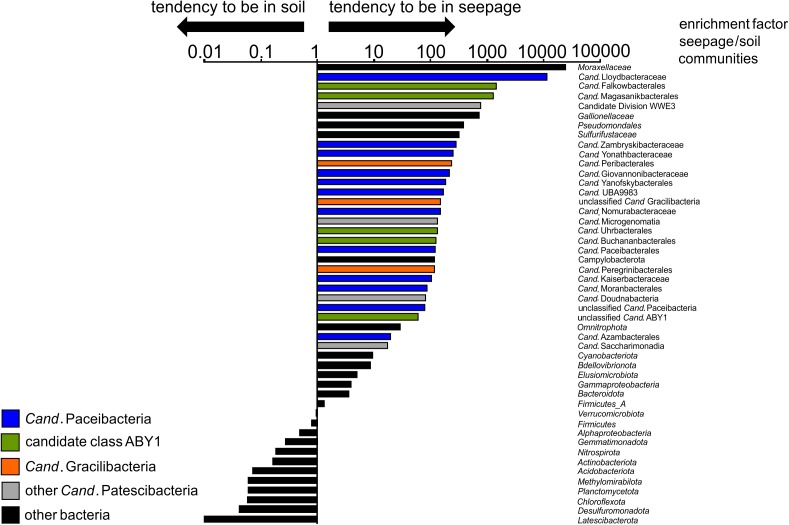
Enrichment factors in seepage compared to soil for selected bacterial taxa (order/family level for *Cand*. Patescibacteria, class level for *Proteobacteria*, phylum level for other Bacteria). For calculation of these factors, mean values of relative abundances of a given taxon across all seepage samples from four locations and different time points (*n* = 10) were divided by mean values of relative abundances of the same taxon across all forest soil samples (*n* = 10). Relative abundances of taxa are based on 16S rRNA gene-targeted Illumina MiSeq amplicon sequencing.

Surprisingly, members of *Cand*. Patescibacteria already dominated seepage from top soil horizons (30 cm depth), with relative abundances up to 50% (mean: 36 ± 12%, *n* = 10, Figure 4A and Supplementary Figure 1), although they represented only 0.55% (±0.34, *n* = 10) of the total bacterial community in forest soil. Likewise, abundances of 21 to 40% (mean: 30 ± 6%, *n* = 15) were observed throughout the lower aquifer assemblage. In addition to *Cand*. Patescibacteria, *Nitrospirota* became the most abundant group in the groundwater, although being only rarely present in soil and seepage (Supplementary Figure 1). *Acidobacteriota*, *Actinobacteriota*, and *Planctomycetota*, which made up more than 37% of the soil bacterial community, were either not mobilized into the seepage at all, or in low abundance, and their relative abundances decreased substantially in the groundwater (Supplementary Figure 1A). Enrichment factors in seepage compared to soil for *Alpha*- and *Gammaproteobacteria* were one to two orders of magnitude lower than for members of *Cand*. Patescibacteria. Nevertheless, they still formed a stable fraction in seepage and groundwater communities (Figure 5 and Supplementary Figure 1A). Overall, 9.5% of the species-level OTUs in soil - assigned using a 97% sequence identity cut-off - were shared with seepage. The strong enrichment of *Cand*. Patescibacteria in the seepage compared to soil was confirmed when following species-level OTUs, revealing enrichment factors higher than 100 especially for OTUs affiliated with *Cand*. Nomurabacteraceae, *Cand*. Kaiserbacteraceae, and unclassified UBA9983 at the family level (Supplementary Figure 5). Altogether, these findings suggested a preferential mobilization and vertical transport of *Cand*. Patescibacteria from soils into the subsurface, confirming our assumptions that soils of the preferential recharge area are an important source of these organisms. In the next step, we aimed to elucidate how the mobilized organisms were thriving in the groundwater, and which parameters influenced their distribution patterns.

### Differentiation of Groundwater *Cand*. Patescibacteria Communities

Along the sloping bedrock strata of the Hainich CZE, the groundwater microbiome can be sampled via several observation wells along a 5.4 km horizontal transect, from 5.1 m (well H13) down to 88 m (well H51) below the surface within a connected aquifer assemblage. The wells H13 to H51 represent an increase in lateral distance to the respective preferential recharge areas, assuming a higher surface-connectivity of the high-permeability strata of this aquifer assemblage that outcrop at uphill positions (Kohlhepp et al., 2017). Interestingly, *Cand*. Paceibacteria and candidate class ABY1, the dominant *Cand*. Patescibacteria classes, showed divergent distributions along the aquifer assemblage (Figure 4B). *Cand*. Paceibacteria consistently made up one to two thirds of the *Cand*. Patescibacteria community in soil and seepage, respectively, and about 50% in groundwater from well H13 to downstream positions (well H51). Candidate class ABY1, however, were barely detected in soil and seepage but increased continuously in relative abundance in groundwater with increasing distance to the hilltop preferential recharge area. In the deepest well, H51, candidate class ABY1 made up 25% of the *Cand*. Patescibacteria community. Similarly, we observed an increase in the fraction of *Cand*. Gracilibacteria from less than 1% in soil and seepage to 5.7% in well H13 and 10.8% in downstream well H51. However, not all *Cand*. Patescibacteria mobilized from soil were able to thrive in the groundwater. *Cand*. Saccharimonadia dominated in soil and were still abundant in seepage, but decreased continuously along the groundwater transect (Figure 4B). These organisms are able to metabolize sugar compounds under oxic and anoxic conditions and in association with plant tissue (Albertsen et al., 2013; Kindaichi et al., 2016; Beckers et al., 2017), and hence might be more adapted to soils or near-surface habitats.

To elucidate the mechanisms driving *Cand*. Patescibacteria distribution patterns, we analyzed the total microbial community structure from soil, seepage, and all groundwater wells based on species-level OTUs, confirming that selected OTUs can be traced from forest soil or forest seepage to the groundwater of the lower aquifer assemblage. Their relative abundances increased from less than 0.001% in forest soil or seepage to 4.6% in the groundwater of well H13 (Otu000184, *Cand*. Nomurabacteraceae) or to 6.9 and 2.8% in wells H41 and H51 (Otu000001, *Cand*. Kaiserbacteraceae) (Supplementary Figure 6). In contrast, other soil-derived OTUs were also constantly detectable in the groundwater but at much lower relative abundances (Otu000012, *Cand*. Kaiserbacteraceae; Otu000014, *Cand*. Nomurabacteraceae). Groundwater at well H13 still shared about 35% of *Cand*. Patescibacteria-affiliated OTUs with seepage, while this fraction decreased to 6.6% at well H51 (Supplementary Figure 7). Seepage-derived OTUs contributed major parts of the *Cand*. Patescibacteria community, with 71.4% relative abundance at the hill top position (H13), contributing 23.5% to the total microbial community. Even at well H51, they formed 44.4% of the *Cand*. Patescibacteria community and 13.7% of the total microbial community (Supplementary Figure 7). In contrast, the shared fraction was lower between soil and groundwater (Supplementary Figure 7), probably due to the fact that only a low fraction of the soil OTUs was mobilized with seepage. Moreover, some potentially shared *Cand*. Patescibacteria OTUs may have been overlooked given the overall low fraction of *Cand*. Patescibacteria in the soil communities.

Following the models of Stegen et al. (2013, 2015), we estimated the contribution of different mechanisms to the formation of the groundwater microbiomes of the lower aquifer assemblage. Stochastic processes played a dominant role for bacterial community assembly, as indicated by β-nearest taxon indices between −0.61 and 1.63 for all pairwise comparisons between bacterial communities in the groundwater wells. The RC_bray_ values for all pairwise comparisons were 1, indicating that dispersal limitation, and hence impediment of the transport of microbes between wells, was the primary mechanism influencing community assembly. Due to the uniform hydrochemistry of the lower aquifer assemblage, community assembly was not driven by variable selection, i.e., the selective influence of environmental parameters on different microbial groups. The multi-story subsurface architecture of the Hainich CZE provides several distinct clusters of groundwater chemistry (Kohlhepp et al., 2017), from oxic to anoxic conditions, which support a high metabolic diversity of the groundwater microbial communities. Hence, we extended our analysis to both aquifer assemblages, to explore whether hydrochemical preferences were driving the differentiation of the *Cand*. Patescibacteria community.

**FIGURE 6:**
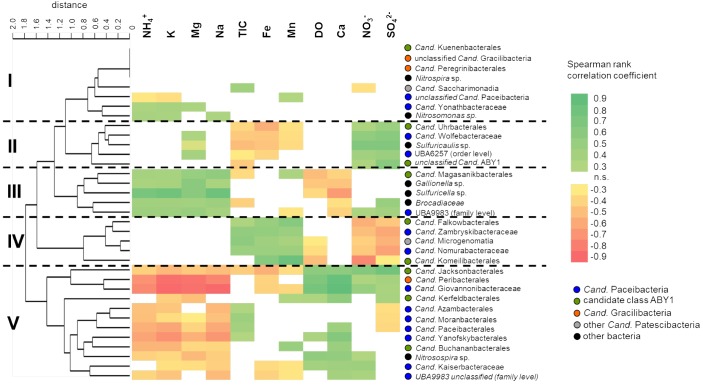
Correlation of selected class-, order- and family- level bacterial groups with environmental parameters across the two aquifer assemblages. Correlations were calculated using Spearman rank correlation coefficients (r_S_) with acceptance of significance at *p* < 0.05, *n* = 46. Colors indicate strength of correlation from r_S_ = 0.9 to r_S_ = –0.9. Taxa were clustered according to similar patterns in their correlation with environmental parameters using paired group algorithm (UPGMA) and Euclidean similarity index. DO, dissolved oxygen; TIC, total inorganic carbon.

**FIGURE 7:**
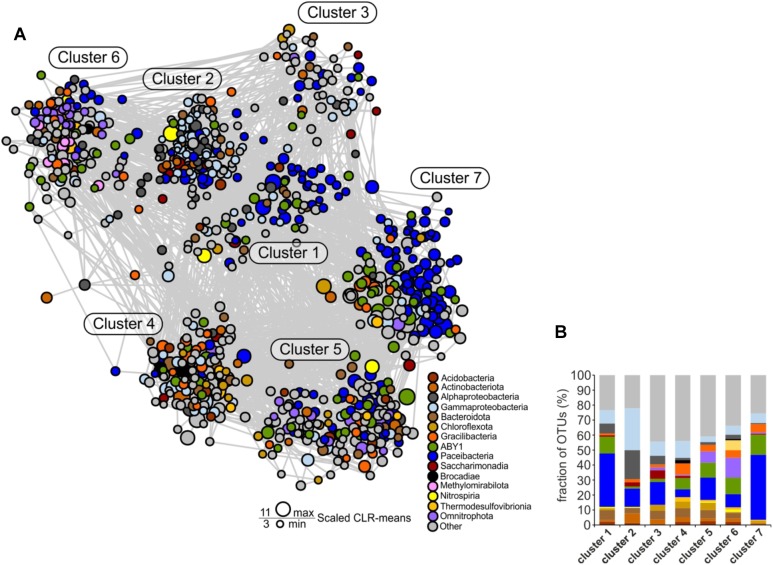
Co-occurrence network of species-level OTUs across the two aquifer assemblages. For each sample, data sets from the 0.2 and 0.1 μm filter size fraction were merged. Only significant edges are displayed. **(A)** Co-occurrence network. Construction was based on a centered log-ratio (CLR) transformed OTU table and Meinshausen-Bühlmann neighborhood selection as graphical inference model. **(B)** Detailed view of the relative fraction of taxonomic groups across the seven clusters of the network.

### Effect of Hydrochemical Parameters on *Cand*. Patescibacteria Community Differentiation

To identify their hydrochemical preferences, we correlated relative abundances of different *Cand*. Patescibacteria taxa with environmental parameters. Five distinct clusters of *Cand*. Patescibacteria taxa were observed based on the obtained correlations, showing no class-specific separation (Figure 6). These clusters showed widely contrasting preferences with regard to the concentration of nitrate and sulfate, as well as ammonium, sodium, potassium, and magnesium. Interestingly, we also observed positive correlations with oxygen concentration for *Cand*. Kaiserbacteraceae (*Cand*. Paceibacteria), *Cand*. Jacksonbacterales (ABY1) and further taxa, while *Cand*. Nomurabacteraceae (*Cand*. Paceibacteria), *Cand*. Komeilibacterales, and *Cand*. Magasanikibacterales (both ABY1) showed negative correlations with oxygen. Concentrations of TOC were usually less than 3 mg L^−1^ and showed only minor spatiotemporal variation across the two aquifer assemblages, yielding mostly non-significant correlations with the bacterial taxa included here (data not shown).

We further included autotrophic bacteria involved in key processes in our groundwater, like nitrification, sulfur and iron oxidation, and anammox (Herrmann et al., 2015; Kumar et al., 2017; Schwab et al., 2017) in our analysis. Interestingly, *Cand*. Magasanikibacterales showed similar correlation patterns to anammox-performing *Brocadiaceae*, iron-oxidizing *Gallionella* and thiosulfate-oxidizing *Sulfuricella*, while *Cand*. Kaiserbacteraceae shared hydrochemical preferences with ammonia-oxidizing *Nitrosospira*. These common preferences for environmental conditions indicate a co-localization of *Cand*. Patescibacteria and key autotrophs along the well transect. With their reduced genomes and limited biosynthetic capabilities, *Cand*. Patescibacteria depend on the uptake of nucleotides and amino acids from co-localized organisms (Brown et al., 2015). The presence of these autotrophs hence might be another factor driving the distribution patterns of *Cand*. Patescibacteria.

### Support for a Central Role of *Cand*. Patescibacteria in Community Networks

To identify potential associations of *Cand*. Patescibacteria with specific bacterial taxa across the two aquifer assemblages, we performed co-occurrence network analysis. The high complexity of interactions present in the diverse groundwater microbial communities was reduced by restricting the analysis to 854 OTUs that were represented by at least 100 sequence reads across all samples. These OTUs represented 68% of the total sequence reads of all the groundwater samples. Moreover, we focused on positive correlations with edge confidence above a cutoff of 0.5 only (Supplementary Figure 8).

All seven distinct clusters of OTUs revealed by the network analysis contained members of *Cand*. Patescibacteria, pointing out their central role in the groundwater microbial communities (Figure 7A). Especially in clusters 1 and 7, *Cand*. Patescibacteria OTUs were dominant, constituting 40 and 70% of all OTUs, respectively (Figure 7A,B). The most abundant families in these clusters were *Cand*. Kaiserbacteraceae and *Cand*. Nomurabacteraceae of the order *Cand*. Paceibacteria. Compared to the other *Cand*. Patescibacteria classes, *Cand*. Paceibacteria co-occurred more often with *Cand*. Patescibacteria-affiliated OTUs. In contrast, first-degree neighbors of candidate class ABY1, *Cand*. Gracilibacteria, and *Cand*. Saccharimonadia included a significantly larger fraction of non-Patescibacteria OTUs (Mann–Whitney *U* test, *p* = 0.0025, Supplementary Figure 9).

As previous studies suggested an important role of autotrophic microorganisms in the groundwater of the Hainich CZE (Herrmann et al., 2015; Kumar et al., 2017; Nowak et al., 2017; Schwab et al., 2017), we were specifically interested in co-occurrence patterns that would suggest specific interactions between *Cand*. Patescibacteria and autotrophs. The putative nitrifiers, anammox bacteria, as well as iron and thiosulfate oxidizers in the groundwater co-occurred more frequently with OTUs affiliated with candidate class ABY1 or *Cand*. Gracilibacteria than with *Cand*. Paceibacteria. First-degree neighbors of putative autotrophs included 22 and 35% of all ABY1 or *Cand*. Gracilibacteria-affiliated OTUs in the network but only 13% of *Cand*. Paceibacteria-affiliated OTUs.

**FIGURE 8:**
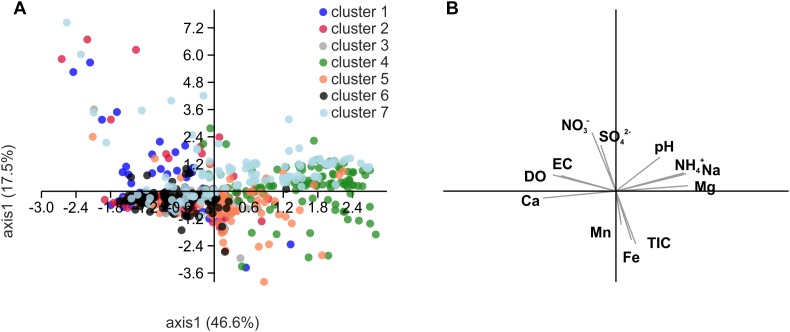
Scatter plot derived from principal component analysis (PCA) of Spearman rank correlation coefficients of selected hydrochemical parameters with relative abundances of the OTUs included in the network of Figure 7 **(A)** and biplot of the correlated hydrochemical parameters **(B)**. To test for an effect of chemical parameters on the association of OTUs in the identified subclusters of the network, we performed pairwise correlations of relative abundances of each OTU with hydrochemical parameters and then subjected the resulting correlation coefficients of significant correlations to PCA. Correlation coefficients were calculated with relative abundances of OTUs from ten groundwater wells and one (H13, H14), two (H31) or six sampling time points per well (total *n* = 46). DO, dissolved oxygen; TIC, total inorganic carbon.

The segregation of the seven clusters resulting from OTU co-occurrence patterns could only marginally be explained based on the correlation of these OTUs with hydrochemical parameters (Figure 8; Spearman rank correlation coefficient, r_S,_ calculated individually for each OTU). Clusters 4 and 6 seemed to be partially driven by ammonium concentration (cluster 4: mean r_S_ calculated from all OTUs of that cluster = 0.50 ± 0.13) as well as oxygen and calcium concentrations (cluster 6: r_S_ (oxygen) = 0.37 ± 0.15; r_S_ (calcium) = 0.46 ± 0.12), but no further correlations driving the differentiation of other clusters were observed.

## Discussion

Microbiomes in subsurface habitats, from shallow aquifers to deep continental crystalline rocks, are typically characterized by high abundance of members of *Cand*. Patescibacteria and by ultra-small cell sizes (Luef et al., 2015; Hubalek et al., 2016; Proctor et al., 2018). So far, little is known about the formation of subsurface microbiomes and about the origin of *Cand*. Patescibacteria in groundwater. An autochtonous (syn-) sedimentary origin and long-term descent of these organisms in our marine bedrock (Middle Triassic) is unlikely, since rock core analyses of the Hainich CZE reveal neither endolithic *Cand*. Patescibacteria, nor their presence on rock fractures (Lazar et al., 2019). Alternatively, these organisms must be introduced from the surface and establish and maintain stable populations in the groundwater. The infrastructure of the Hainich CZE to sample soil, seepage, and groundwater in the topographic recharge and transit area of the local groundwater flow system allows for the analysis of the introduction of soil-derived microorganisms into the groundwater. Since the strata of the lower aquifer assemblage (HTL) crop out at upper hill areas that are thought to function as preferential surface-recharge areas (Kohlhepp et al., 2017), we followed changes in the groundwater microbiome along the subsurface water flow from its origin through shallow perched groundwater to deeper saturated zones. Seepage-dependent release and transport - including colloid-assisted transport - are passive dispersal mechanisms (Dibbern et al., 2014; Lehmann et al., 2018; Zhang et al., 2018) that link soil, subsoil and groundwater microbiomes, allowing organisms to colonize the groundwater and spread along the multi-story aquifer system of the Hainich CZE. This repetitive flow of water and transport of cells and matter creates a constant disturbance characteristic for shallow groundwater ecosystems, influencing community assembly. Soil-derived organisms are transported into the groundwater, where they become part of the community present, and can thrive and increase in abundance if encountering suitable conditions. In addition, interactions and exchange between planktonic groundwater microorganisms and those that are attached to aquifer rock may affect the assembly of groundwater microbial communities. In fact, recent investigations at our study site suggested considerable mobilization and (re)dispersal of attached microorganisms, as more than 44% of rock matrix-associated genera were also found in the groundwater (Lazar et al., 2019). Interestingly, *Cand*. Patescibacteria accounted for less than 1% of the rock matrix community (Lazar et al., 2019), suggesting that their distribution in the two aquifer assemblages might be less affected by interactions between planktonic and attached populations.

Here we show that *Cand*. Patescibacteria make up a large part of this microbial input into the groundwater. Forested soils in the hilltop preferential recharge area are the most probable source of *Cand*. Patescibacteria at our study site, as these organisms were mobilized in high abundance in seepage, despite their low relative abundance in the soil microbial communities. Likewise, Zhang et al. (2018) recently observed high abundance of *Cand*. Patescibacteria in seepage collected beneath maize-planted agricultural soils, indicating preferential mobilization as a common trait for these organisms. Surface charge of cells, hydrophobicity, or cell surface macromolecules have been discussed as factors influencing transport of bacteria in porous media (Wan et al., 1994; Bolster et al., 2009; Kim et al., 2009). *Cand*. Patescibacteria encode large cell surface proteins, most likely involved in the attachment to other microorganisms (Castelle et al., 2018). However, a potential supporting effect of these surface proteins on the mobilization by percolating water in soils remains currently unclear. The fact that surface charge is negative for soil mineral particles and is most likely also negative for *Cand*. Patescibacteria cells, as it holds for most microorganisms (Koyama et al., 2013), could contribute to generally favorable conditions for cell dispersal in soil. The low ionic strengths observed in soil seepage (<2 mmol L^−1^) reduce attachment (Wang et al., 2013), further promoting microbial dispersal. In the saturated zone, ionic strengths range from 5 (shallow perched groundwater) to ∼20 mmol L^−1^ (deepest well), pointing to variable but also unfavorable conditions for the subsurface mobility of microbial cells. This reduced mobility, together with the heterogeneous structure of the fractured aquifer rock, might explain why the compositional turnover of microbial communities across the lower aquifer assemblage was dominated by dispersal limitation.

The Hainich CZE provides several distinct hydrochemical zones on a single hillslope, differing strongly in concentrations of oxygen, nitrogen compounds, iron, and sulfur compounds and hence supporting a high functional diversity of microorganisms. Consequently, when investigating the distribution patterns of *Cand*. Patescibacteria, we observed a high and taxon-specific variability both for correlations with environmental parameters and co-occurrence with putative partner organisms. Especially the distribution patterns of *Cand*. Paceibacteria appeared to be independent of specific partners, as they primarily showed interconnections among themselves and to other *Cand*. Patescibacteria. The contrasting preferences for hydrochemical conditions among several groups of these organisms prevented general conclusions about parameters driving their distribution. The fermentative metabolism postulated for some members of the *Cand*. Paceibacteria (Brown et al., 2015; Nelson and Stegen, 2015) would provide independence of inorganic electron acceptors, and could explain their ubiquitous predominance throughout the groundwater flow system. The availability of resources not targeted in this study, such as essential organic monomers many *Cand*. Paceibacteria are not able to synthesize themselves due to the lack of metabolic pathways (Brown et al., 2015), might be a stronger driver of their distribution. The high abundance of transporter and glycoside hydrolase genes described (Brown et al., 2015; Castelle et al., 2017; Danczak et al., 2017), together with the high surface-to-volume ratio of ultra-small cells, can be seen as optimizations for the uptake of the low concentrations of such compounds in the oligotrophic groundwater.

Previous studies suggested that most *Cand*. Patescibacteria are anaerobes based on the lack of respiratory chains (Castelle et al., 2018). However, we found surprisingly high abundances of *Cand*. Patescibacteria in oxic groundwater. Moreover, the distribution patterns of the abundant *Cand*. Kaiserbacteraceae, *Cand*. Giovannonibacteraceae, and *Cand*. Nomurabacteraceae across the two aquifer assemblages pointed to contrasting preferences for oxic or anoxic conditions, suggesting differences in the potential utilization of electron acceptors besides a fermentative life style, or in the ability to deal with oxidative stress (Léon-Zayas et al., 2017). Similarly, the presence of nitrite reductase encoding genes in genomes of *Cand*. Patescibacteria has been ascribed to nitrite detoxification mechanisms rather than anaerobic respiration or denitrification (Castelle et al., 2018).

In contrast to *Cand*. Paceibacteria, candidate class ABY1 and *Cand*. Gracilibacteria shared a higher number of positive correlations with taxonomic groups other than *Cand*. Patescibacteria in our network analysis, suggesting that these groups shared ecological niches with other bacterial groups or showed a higher level of dependency on the positively correlated taxa. Interestingly, both classes were also more enriched in the 0.2 μm fraction compared to the majority of order- or family- level taxonomic groups within the *Cand*. Paceibacteria. This might indicate larger cell sizes as previously suggested for *Cand*. Gracilibacteria (”*Cand*. Peregrinibacteria”; Castelle et al., 2018), or could hint to a proclivity for aggregation, including associations with other microorganisms. For *Cand*. Paceibacteria, *Cand*. Gracilibacteria, and candidate class ABY1, network analyses revealed a significant co-occurrence with autotrophic organisms involved in nitrogen, sulfur, and iron cycling. Unfortunately, interpretations on parasitic or symbiotic interactions with these autotrophic taxa remain currently highly speculative: To date, symbiotic interactions have only been experimentally demonstrated for one member of *Cand*. Paceibacteria, *Cand*. Sonnebornia yantaiensis (Gong et al., 2014), which is part of a three-member consortium including an autotrophic partner (*Chlorella*). Given the high relative abundance of some autotrophic groups in the groundwater of the Hainich CZE (Herrmann et al., 2015; Kumar et al., 2017), potential direct interactions would have strong implications for subsurface carbon cycling, as part of the carbon fixed by autotrophy would be shuffled through the abundant *Cand*. Patescibacteria biomass.

## Conclusion

In conclusion, we propose key mechanisms leading to the success of *Cand*. Patescibacteria in groundwaters. Our novel findings demonstrate: (1) specific order- and family- level groups within *Cand*. Patescibacteria are preferentially mobilized from soils into the groundwater, (2) where they ultimately increase in relative abundance and become the dominant microbial groups, and finally (3) we suggest that spatial differentiation of *Cand*. Patescibacteria in the groundwaters of the Hainich CZE is driven by hydrochemical parameters, resource availability supporting a fermentative lifestyle, as well as interactions with and potential dependence on other bacterial taxa, including abundant autotrophic groups.

## Author Contributions

MH and KK designed this study. KT and KK designed the Hainich Critical Zone Exploratory. KT, RL, and KL established field infrastructure and provided groundwater and seepage samples, and provided hydrochemical data and data of Nanoparticle Tracking Analysis. MH performed most of the molecular work and sequence analysis. CEW carried out co-occurrence network analysis. PG performed the bacterial starvation experiments. MT performed statistical analyses. LY performed the calculation of community assembly mechanisms. MT, MH, and KK wrote the manuscript with contributions from all other authors.

## Conflict of Interest Statement

The authors declare that the research was conducted in the absence of any commercial or financial relationships that could be construed as a potential conflict of interest.

## Footnotes

1 http://gtdb.ecogenomic.org/

2 https://github.com/stegen/Stegen_etal_ISME_2013

3 https://cran.r-project.org/web/packages/Matrix/index.html

4 https://rrndb.umms.med.umich.edu/
