# Supplementary material for: Predominance of Cand. Patescibacteria in Groundwater Is Caused by Their Preferential Mobilization From Soils and Flourishing Under Oligotrophic Conditions
Source: Front Microbiol. 2019 Jun 20;10:1407. doi: 10.3389/fmicb.2019.01407 (PMC6596338; doi:10.3389/fmicb.2019.01407)
Supplement: Supplementary file 1 [file Data_Sheet_1.zip › 1Herrmann_et_al_Supplementary_Material_content.pdf]

Supplementary Material

Predominance of *Cand.* Patescibacteria in groundwater is caused by their preferential mobilization from soils and flourishing under oligotrophic conditions

Martina Herrmann[1,2], Carl-Eric Wegner[1], Martin Taubert[1], Patricia Geesink[1], Katharina Lehmann[3], Lijuan Yan[1,2], Robert Lehmann[3], Kai Uwe Totsche[3], Kirsten Küsel[1,2*]

*** Correspondence: Kirsten Küsel:** kirsten.kuesel@uni-jena.de

Supplementary Material contains:

Supplementary Figure 1

Supplementary Figure 2

Supplementary Figure 3

Supplementary Figure 4

Supplementary Figure 5

Supplementary Figure 6

Supplementary Figure 7

Supplementary Figure 8

Supplementary Figure 9

Supplementary Table 1

Supplementary Table 2
