# Supplementary material for: Predominance of Cand. Patescibacteria in Groundwater Is Caused by Their Preferential Mobilization From Soils and Flourishing Under Oligotrophic Conditions
Source: Front Microbiol. 2019 Jun 20;10:1407. doi: 10.3389/fmicb.2019.01407 (PMC6596338; doi:10.3389/fmicb.2019.01407)
Supplement: Supplementary file 1 [file Data_Sheet_1.zip › Herrmann_et_al_Supplementary_Figure1.pdf]

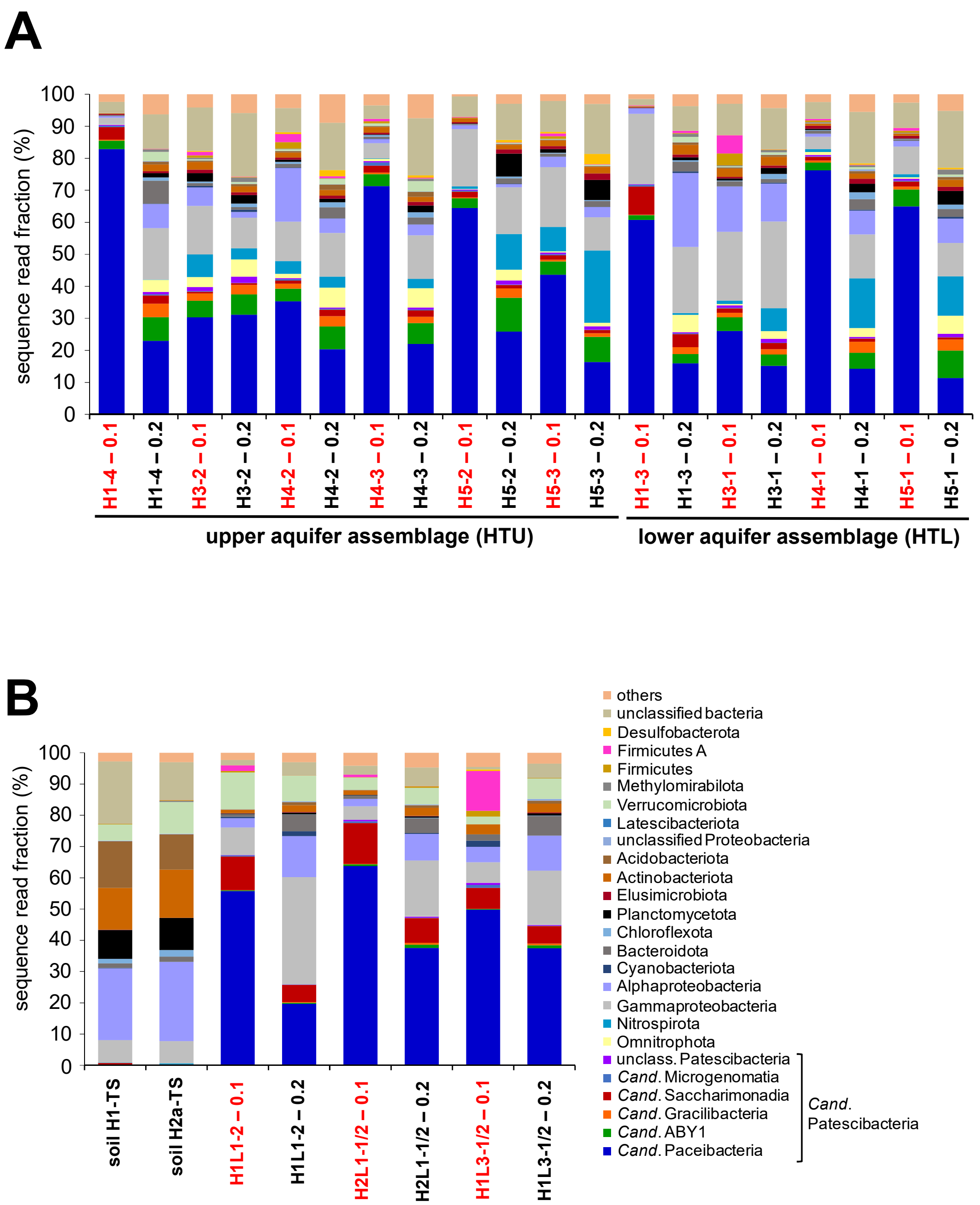

Supplementary Figure 1. Bacterial community structure in the 0.1 µm filter fraction (labeled in red) and 0.2 µm filter fraction (labeled in black) of groundwater of the two aquifer assemblages (A) and forest seepage (B). Community structure of forest soil was added to (B) for comparison. For groundwater, data are means of two (H31) or six sampling time points per site. For well H13 and H14, data are only available for one time point. For soil, data are means (H1-TS, H2a-TS) of five spatial replicates. H1L1-2, H2L1-1/2, H1L3-1/2: seepage collected at 30 cm soil depth. Lysimeter data originate from one sampling time point except for H1L1-2 (n=6).
