# Supplementary material for: Predominance of Cand. Patescibacteria in Groundwater Is Caused by Their Preferential Mobilization From Soils and Flourishing Under Oligotrophic Conditions
Source: Front Microbiol. 2019 Jun 20;10:1407. doi: 10.3389/fmicb.2019.01407 (PMC6596338; doi:10.3389/fmicb.2019.01407)
Supplement: Supplementary file 1 [file Data_Sheet_1.zip › Herrmann_et_al_Supplementary_Figure2.pdf]

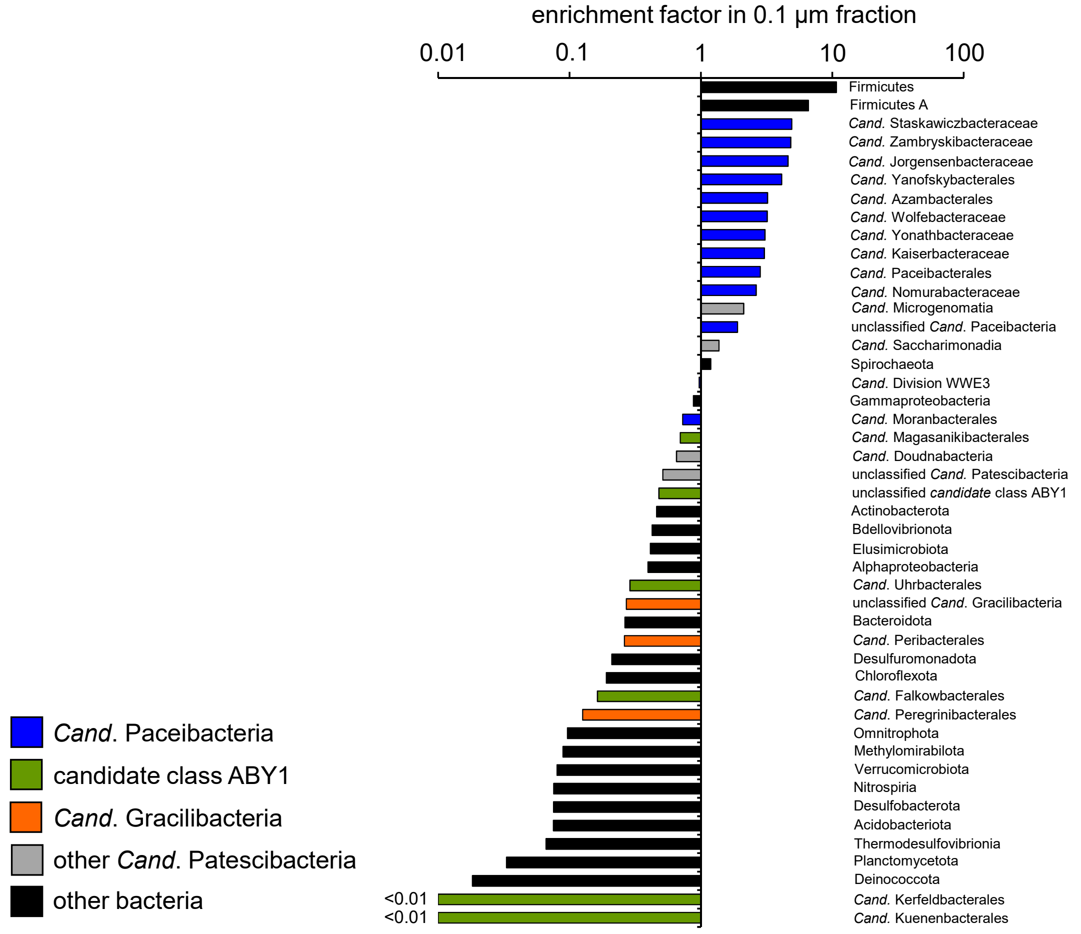

Supplementary Figure 2. Enrichment factor of selected bacterial taxa (class/order/family level for *Cand*. Patescibacteria, class level for *Proteobacteria*, phylum level for other Bacteria) in the 0.1 µm filter fraction. Enrichment factors were assessed by comparing relative abundances of phyla between 0.1 and 0.2 µm filter fractions based on MiSeq Illumina 16S rRNA-gene targeted amplicon sequencing (median values of 10 groundwater wells and one (H13, H14), two (H31) or six sampling time points per well; total n=46).
