# Supplementary material for: Predominance of Cand. Patescibacteria in Groundwater Is Caused by Their Preferential Mobilization From Soils and Flourishing Under Oligotrophic Conditions
Source: Front Microbiol. 2019 Jun 20;10:1407. doi: 10.3389/fmicb.2019.01407 (PMC6596338; doi:10.3389/fmicb.2019.01407)
Supplement: Supplementary file 1 [file Data_Sheet_1.zip › Herrmann_et_al_Supplementary_Figure3.pdf]

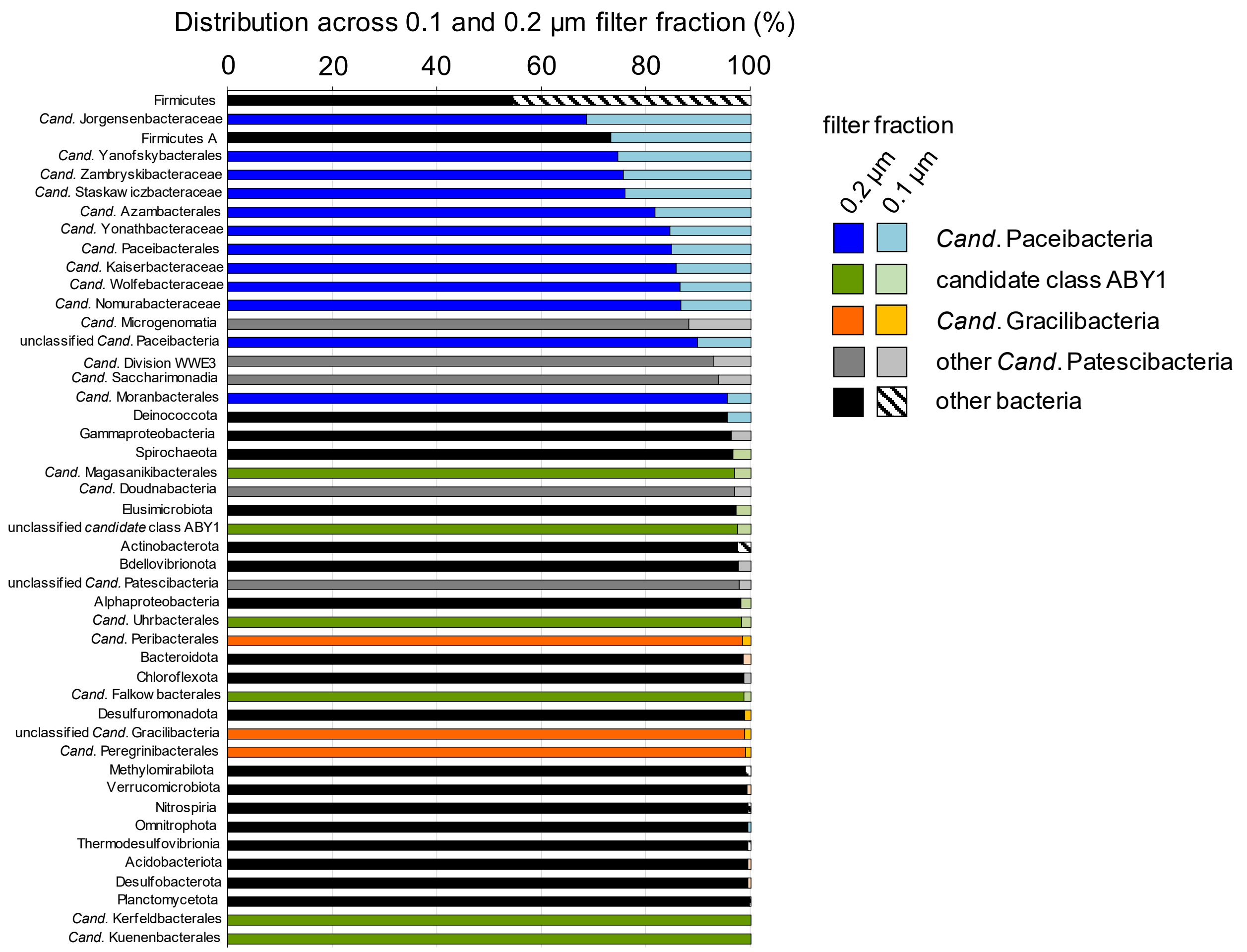

Supplementary Figure 3. Distribution of family/order level taxa (*Cand.* Paceibacteria, ABY1, and Gracilibacteria) or class/phylum level across the 0.1 µm and 0.2 µm filter fractions. Bar charts show which fraction within a given taxon was found in the 0.1 µm filter fraction and the 0.2 µm filter fraction, respectively. Data are median values of 46 samples per filter fraction.
