# Supplementary material for: Predominance of Cand. Patescibacteria in Groundwater Is Caused by Their Preferential Mobilization From Soils and Flourishing Under Oligotrophic Conditions
Source: Front Microbiol. 2019 Jun 20;10:1407. doi: 10.3389/fmicb.2019.01407 (PMC6596338; doi:10.3389/fmicb.2019.01407)
Supplement: Supplementary file 1 [file Data_Sheet_1.zip › Herrmann_et_al_Supplementary_Figure4.pdf]

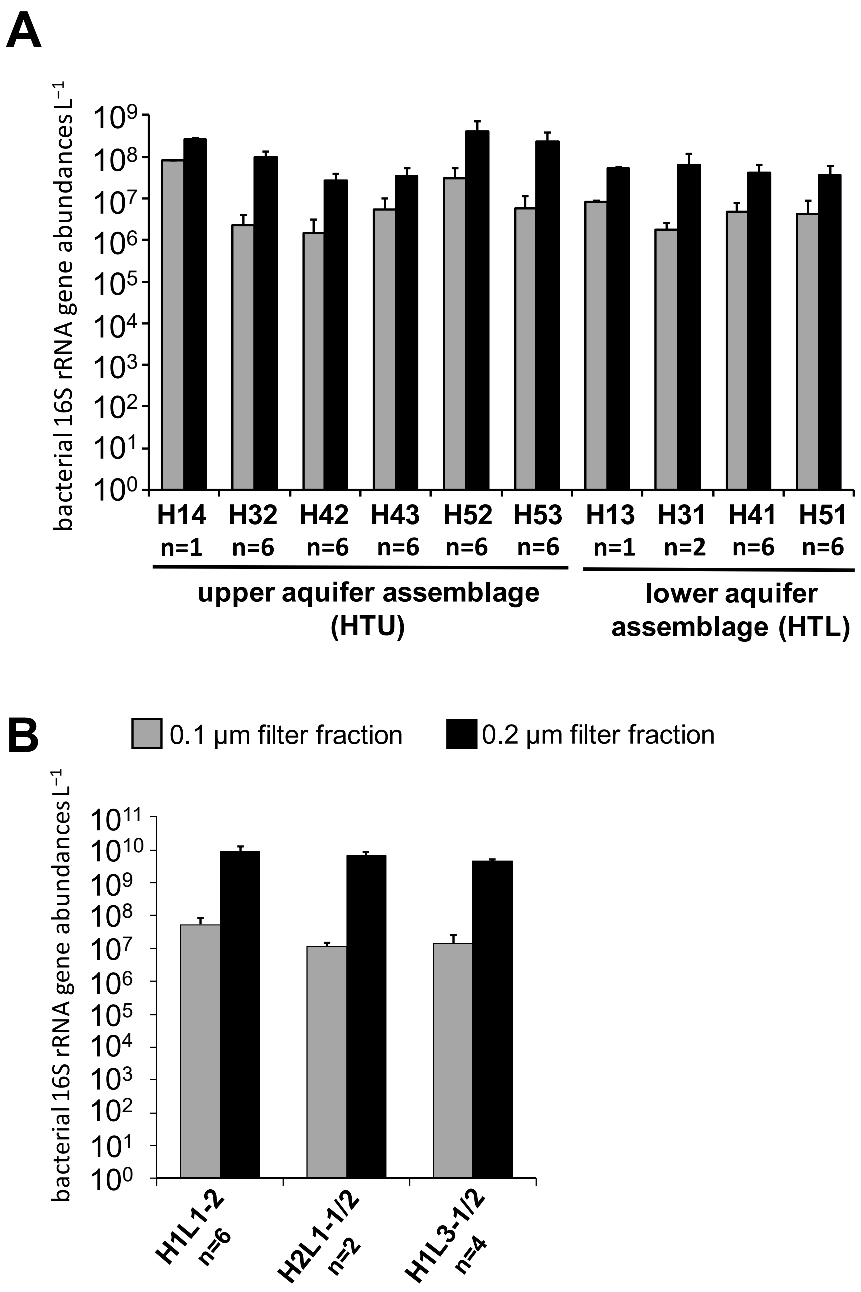

Supplementary Figure 4. Abundances of bacterial 16S rRNA genes in groundwater (A) and forest seepage (B). Black bars represent the 0.2 µm filter fraction, grey bars the 0.1 µm filter fraction. Bars represent mean (± standard deviation) of one sampling time point (H13, H14), two sampling time points (H31) and six sampling time points (all other wells), each run in three technical replicates, for the groundwater samples. For the seepage samples, bars represent mean (± standard deviation) of six sampling time points (H1L1-2), and two spatial replicates (H2L1-1/2) or two spatial replicates with each two sampling time points; H1L3-1/2), each also run in three technical replicates.
