# Supplementary material for: Predominance of Cand. Patescibacteria in Groundwater Is Caused by Their Preferential Mobilization From Soils and Flourishing Under Oligotrophic Conditions
Source: Front Microbiol. 2019 Jun 20;10:1407. doi: 10.3389/fmicb.2019.01407 (PMC6596338; doi:10.3389/fmicb.2019.01407)
Supplement: Supplementary file 1 [file Data_Sheet_1.zip › Herrmann_et_al_Supplementary_Figure5.pdf]

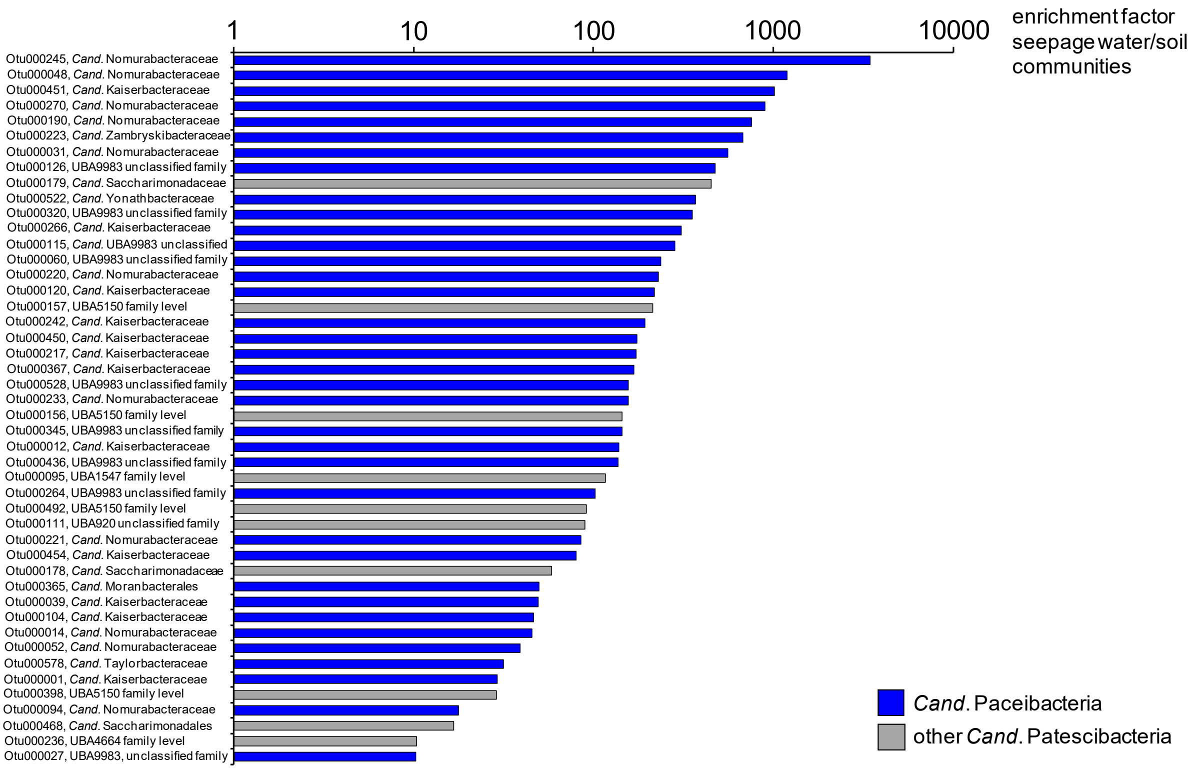

Supplementary Figure 5. Enrichment factors in seepage compared to soil for the first 45 most abundant *Cand*. Patescibacteria OTUs (taxonomically classified on order/family level). For calculation of these factors, mean values of relative abundances of a given taxon across all seepage samples from three locations and different time points (n=10) were divided by mean values of relative abundances of the same taxon across all forest soil samples (n=10). Relative abundances of taxa for these calculations are based on 16S rRNA gene-targeted Illumina MiSeq amplicon sequencing.
