# Supplementary material for: Predominance of Cand. Patescibacteria in Groundwater Is Caused by Their Preferential Mobilization From Soils and Flourishing Under Oligotrophic Conditions
Source: Front Microbiol. 2019 Jun 20;10:1407. doi: 10.3389/fmicb.2019.01407 (PMC6596338; doi:10.3389/fmicb.2019.01407)
Supplement: Supplementary file 1 [file Data_Sheet_1.zip › Herrmann_et_al_Supplementary_Figure6.pdf]

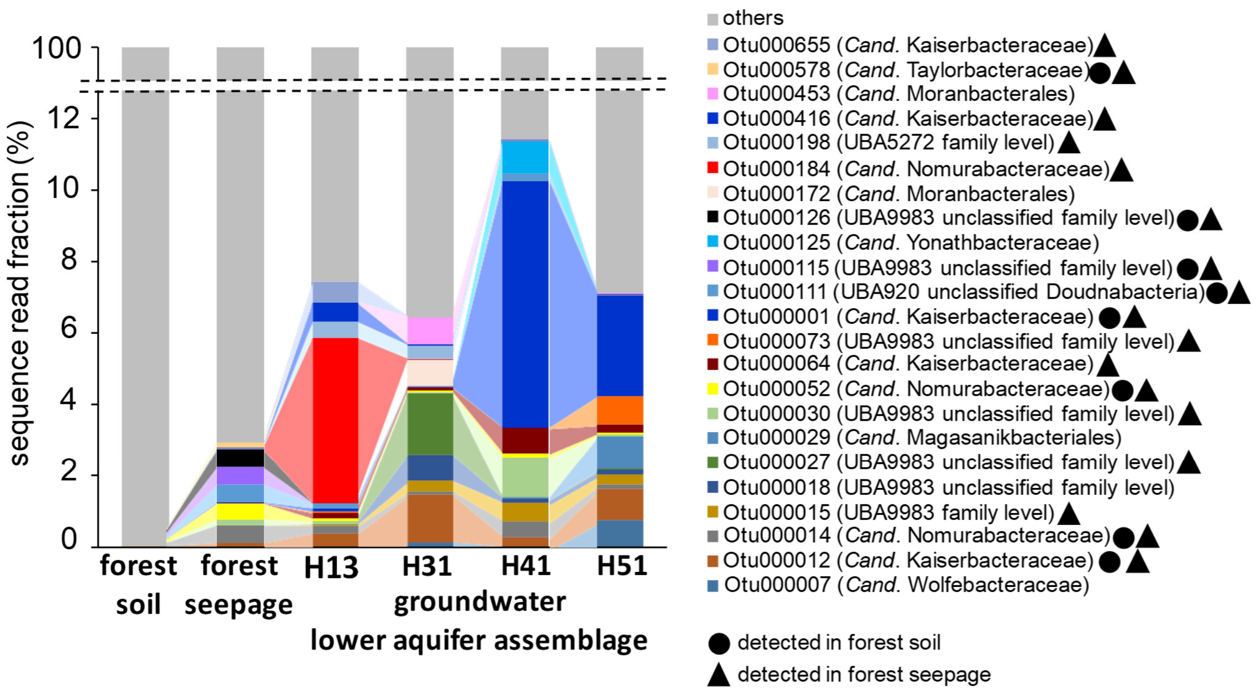

Supplementary Figure 6. Changes in relative abundances of selected *Cand*. Patescibacteria OTUs within the total bacterial community from forest soil and forest seepage to groundwater across the lower aquifer assemblage (wells H13, H31, H41, H51). Results are based on 16S rRNA gene targeted amplicon sequencing. OTUs detected in soil are labeled with filled circles, OTUs detected in forest seepage are labeled with filled triangles. Selection of OTUs targeted the two most abundant OTUs per site. Data represent one time point (H13) or are means of two (H31), six (H41, H51) time points, 10 spatial replicates (forest soil), and 10 temporal and/or spatial replicates (forest seepage).
