# Supplementary material for: Predominance of Cand. Patescibacteria in Groundwater Is Caused by Their Preferential Mobilization From Soils and Flourishing Under Oligotrophic Conditions
Source: Front Microbiol. 2019 Jun 20;10:1407. doi: 10.3389/fmicb.2019.01407 (PMC6596338; doi:10.3389/fmicb.2019.01407)
Supplement: Supplementary file 1 [file Data_Sheet_1.zip › Herrmann_et_al_Supplementary_Figure7.pdf]

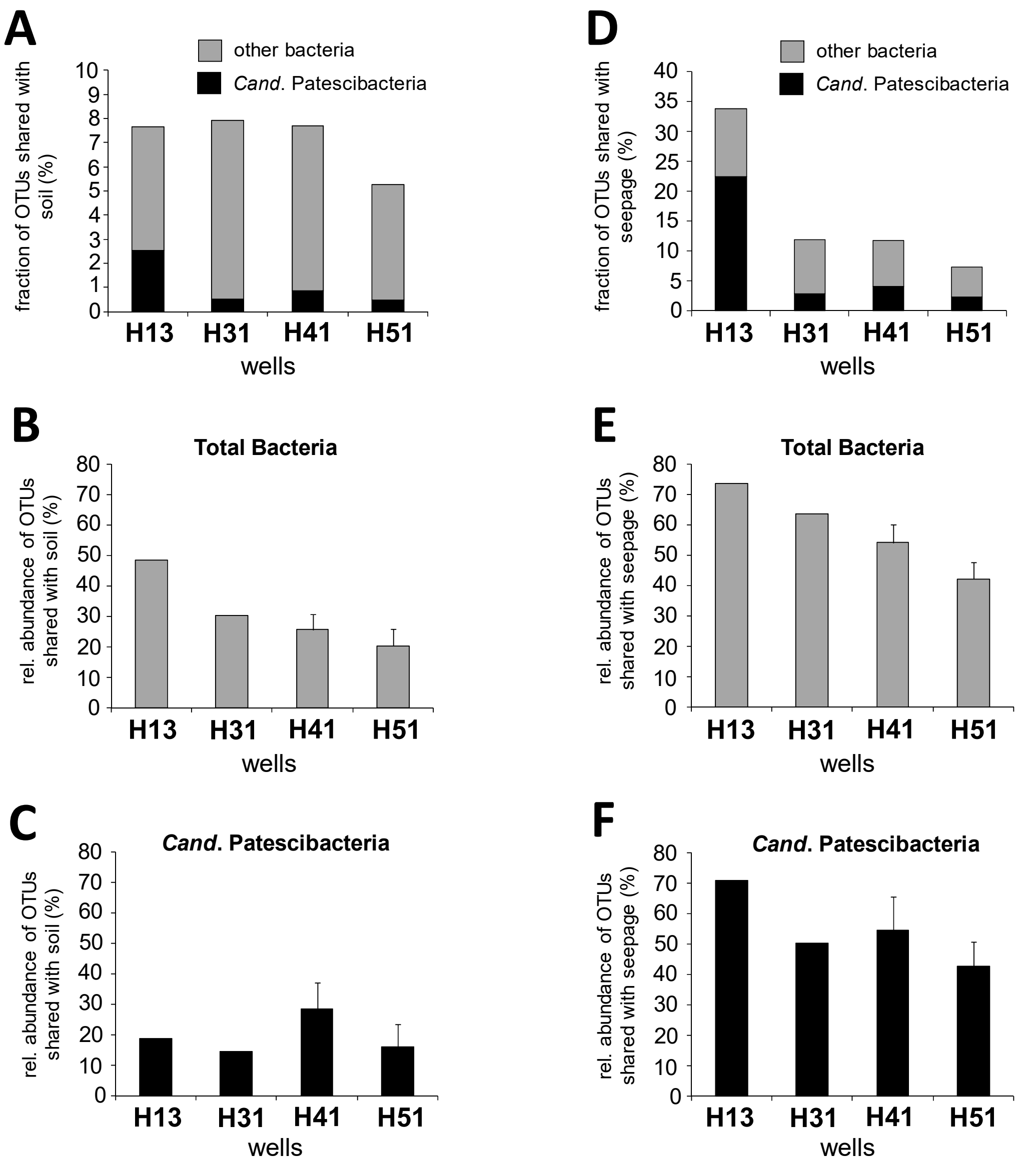

Supplementary Figure 7. Fraction of OTUs and their relative abundance in groundwater at wells H13 to H51 of the lower aquifer assemblage which were shared with forest soil (A-C) or forest seepage (D-F). (A, D) Fraction of shared OTUs within total bacteria, black sections indicate the contribution of *Cand*. Patescibacteria-affiliated OTUs to the shared fraction. (B, E) Relative abundance of all bacterial OTUs in groundwater at wells H13 to H51 which are shared with forest soil or forest seepage. (C, F) Relative abundance of OTUs within the groundwater *Cand*. Patescibacteria community which are shared with forest soil or forest seepage.
