# Supplementary material for: Predominance of Cand. Patescibacteria in Groundwater Is Caused by Their Preferential Mobilization From Soils and Flourishing Under Oligotrophic Conditions
Source: Front Microbiol. 2019 Jun 20;10:1407. doi: 10.3389/fmicb.2019.01407 (PMC6596338; doi:10.3389/fmicb.2019.01407)
Supplement: Supplementary file 1 [file Data_Sheet_1.zip › Herrmann_et_al_Supplementary_Figure9.pdf]

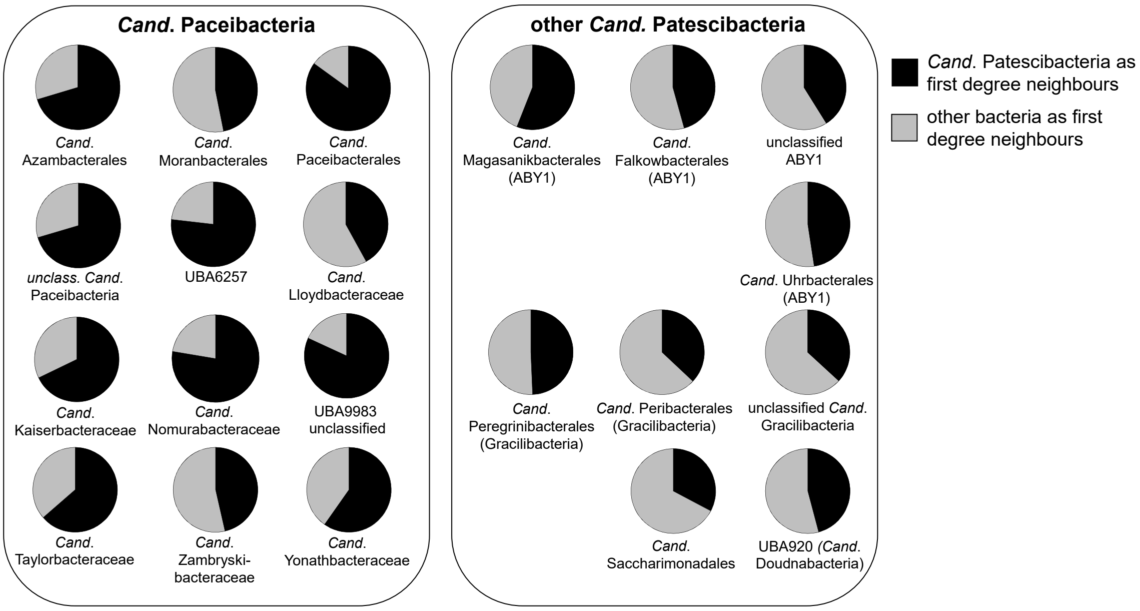


**Supplementary Figure 9.** Fraction of first-degree neighbors in the network that were either affiliated with *Cand*. Patescibacteria or other bacteria. Left panel: Pie charts represent a summary of all first-degree neighbors of OTUs affiliated with different order- and family-level groups within the *Cand*. Paceibacteria. Right panel: Pie charts represent a summary of all first-degree neighbors of OTUs affiliated with other class- and order-level groups within the *Cand*. Patescibacteria.
