# Supplementary material for: Predominance of Cand. Patescibacteria in Groundwater Is Caused by Their Preferential Mobilization From Soils and Flourishing Under Oligotrophic Conditions
Source: Front Microbiol. 2019 Jun 20;10:1407. doi: 10.3389/fmicb.2019.01407 (PMC6596338; doi:10.3389/fmicb.2019.01407)
Supplement: Supplementary file 1 [file Data_Sheet_1.zip › Herrmann_et_al_Supplementary_Table1.pdf]

Supplementary Table 1. Taxonomic classification of the 26 heterotrophic strains isolated from groundwater, which were used for the growth experiments carried out in this study.

isolate name	accession number	taxonomic affiliation (phylum; family)
hainich_062	MG980464	*Bacteroidota*; *Flavobacteriaceae*
hainich_114	MH015183	*Actinobacteriota*; *Nocardioidaceae*
hainich_115	MH015184	*Actinobacteriota*; *Nocardioidaceae*
hainich_001	MG980417	*Bacteroidota*; *Flavobacteriaceae*
hainich_083	MG980482	*Bacteroidota*; *Flavobacteriaceae*
hainich_006	MG980422	*Proteobacteria*; *Caulobacteraceae*
hainich_009	MG980424	Proteobacteria; *Rhodobacteraceae*
hainich_065	MG980465	*Proteobacteria*; *Pseudomonadaceae*
hainich_105	MG980499	*Bacteroidota*; *Flavobacteriaceae*
hainich_007	MH015185	*Bacteroidota*; *Hymenobacteraceae*
hainich_200*	MH015187	*Bacteroidota*; *Flavobacterium*
hainich_053	MG980455	*Proteobacteria*; *Pseudomonadaceae*
hainich_055	MG980457	*Proteobacteria*; *Pseudomonadaceae*
hainich_099	MG980494	*Proteobacteria*; *Neisseriaceae*
hainich_106	MG980500	*Proteobacteria*; *Comamonadaceae*
hainich_108	MH015188	*Proteobacteria*; *Burkholderiaceae*
hainich_110	MH015186	*Proteobacteria*; *Burkholderiaceae*
hainich_119	MG980506	*Proteobacteria*; *Pseudomonadaceae*
hainich_081	MG980480	*Proteobacteria*; *Pseudomonadaceae*
hainich_068	MG980468	*Firmicutes*; *Bacillaceae*
hainich_013	MG980427	*Firmicutes*; *Paenibacillaceae*
hainich_074	MG980474	*Proteobacteria*; *Pseudomonadaceae*
hainich_017	MG980431	*Proteobacteria*; *Pseudomonadaceae*
hainich_023	MG980434	*Proteobacteria*; *Pseudomonadaceae*
hainich_025	MG980436	*Bacteroidota*; *Flavobacteriaceae*
hainich_028	MH015189	*Proteobacteria*; *Moraxellaceae*

*used for TEM analysis
