# Supplementary material for: Predominance of Cand. Patescibacteria in Groundwater Is Caused by Their Preferential Mobilization From Soils and Flourishing Under Oligotrophic Conditions
Source: Front Microbiol. 2019 Jun 20;10:1407. doi: 10.3389/fmicb.2019.01407 (PMC6596338; doi:10.3389/fmicb.2019.01407)
Supplement: Supplementary file 1 [file Data_Sheet_1.zip › Herrmann_et_al_Supplementary_Table2.pdf]

Supplementary Table 2A: Abundances of bacterial 16S rRNA genes per liter in the 0.2 µm and 0.1 µm filter fraction of groundwater samples. Data are means (± s. d.) of three technical replicates. n. a.: no samples available/samples were not analyzed for bacterial 16S rRNA gene abundances.

well	filter fraction	Sep 2015	Nov 2015	Dec 2015	June 2016	Aug 2016	Nov 2016
**H13**	0.2 µm	n. a.	n. a.	n. a.	n. a.	5.4×10^{7} (4.0×10^{6})	n. a.
	0.1 µm	n. a.	n. a.	n. a.	n. a.	8.3×10^{6} (4.6×10^{5})	n. a.
**H14**	0.2 µm	n. a.	n. a.	n. a.	n. a.	2.6×10^{8} (1.6×10^{7})	n. a.
	0.1 µm	n. a.	n. a.	n. a.	n. a.	8.1×10^{7} (3.0×10^{6})	n. a.
**H31**	0.2 µm	n. a.	n. a.	n. a.	1.2×10^{7} (1.0×10^{6})	1.1×10^{8} (4.4×10^{6})	n. a.
	0.1 µm	n. a.	n. a.	n. a.	1.0×10^{6} (1.1×10^{5})	2.5×10^{6} (1.9×10^{5})	n. a.
**H32**	0.2 µm	7.0×10^{7} (6.4×10^{6})	7.6×10^{7} (2.5×10^{6})	6.9×10^{7} (8.5×10^{6})	1.1×10^{8} (1.8×10^{6})	1.3×10^{8} (1.7×10^{7})	1.5×10^{8} (2.2×10^{6})
	0.1 µm	4.1×10^{5} (1.9×10^{4})	1.9×10^{6} (1.3×10^{5})	4.8×10^{6} (1.6×10^{5})	1.9×10^{6} (7.1×10^{4})	3.8×10^{6} (3.3×10^{5})	5.2×10^{5} (6.6×10^{4})
**H41**	0.2 µm	3.3×10^{7} (1.4×10^{6})	3.2×10^{7} (1.9×10^{6})	2.7×10^{7} (1.3×10^{6})	1.6×10^{7} (2.0×10^{6})	8.6×10^{7} (1.2×10^{7})	5.2×10^{7} (4.7×10^{6})
	0.1 µm	1.6×10^{6} (1.3×10^{5})	3.0×10^{6} (2.0×10^{5})	5.8×10^{6} (5.3×10^{5})	1.5×10^{6} (2.1×10^{4})	6.3×10^{6} (4.9×10^{5})	9.5×10^{6} (1.4×10^{5})
**H42**	0.2 µm	2.0×10^{7} (5.7×10^{5})	2.8×10^{7} (5.6×10^{5})	2.5×10^{7} (8.6×10^{5})	1.2×10^{7} (2.8×10^{6})	3.5×10^{7} (7.8×10^{5})	4.4×10^{7} (3.4×10^{6})
	0.1 µm	3.3×10^{5} (1.9×10^{4})	4.8×10^{6} (9.7×10^{4})	3.6×10^{5} (6.7×10^{3})	4.4×10^{5} (7.4×10^{4})	2.2×10^{6} (1.5×10^{5})	8.5×10^{5} (5.1×10^{4})
**H43**	0.2 µm	1.2×10^{7} (1.1×10^{6})	6.3×10^{7} (1.8×10^{6})	4.9×10^{7} (1.5×10^{6})	1.4×10^{7} (4.1×10^{6})	3.0×10^{7} (6.8×10^{5})	4.4×10^{7} (2.5×10^{5})
	0.1 µm	1.4×10^{7} (3.8×10^{4})	1.7×10^{6} (1.9×10^{5})	6.9×10^{6} (5.5×10^{5})	7.1×10^{5} (1.2×10^{5})	3.5×10^{6} (1.2×10^{5})	5.5×10^{6} (3.1×10^{5})
**H51**	0.2 µm	2.1×10^{7} (9.0×10^{6})	2.4×10^{7} (1.6×10^{6})	3.8×10^{7} (1.4×10^{6})	1.4×10^{7} (6.4×10^{5})	6.3×10^{7} (9.4×10^{5})	7.0×10^{7} (3.8×10^{6})
	0.1 µm	1.7×10^{6} (1.9×10^{5})	5.7×10^{6} (3.2×10^{5})	4.2×10^{5} (6.4×10^{3})	2.0×10^{6} (1.7×10^{5})	3.7×10^{6} (2.5×10^{5})	1.3×10^{7} (6.3×10^{5})
**H52**	0.2 µm	1.9×10^{8} (9.4×10^{7})	1.4×10^{8} (1.2×10^{7})	6.6×10^{8} (4.9×10^{7})	6.7×10^{7} (3.0×10^{7})	5.5×10^{8} (2.4×10^{7})	8.6×10^{8} (9.0×10^{7})
	0.1 µm	1.8×10^{7} (4.2×10^{5})	4.4×10^{7} (2.2×10^{6})	5.1×10^{7} (4.0×10^{6})	2.7×10^{6} (2.2×10^{5})	7.4×10^{6} (3.2×10^{5})	6.1×10^{7} (4.2×10^{6})
**H53**	0.2 µm	1.8×10^{8} (3.0×10^{6})	1.2×10^{8} (4.8×10^{6})	1.0×10^{8} (1.8×10^{7})	2.0×10^{8} (2.9×10^{7})	5.1×10^{8} (2.1×10^{7})	3.2×10^{8} (2.2×10^{7})
	0.1 µm	4.1×10^{6} (2.6×10^{5})	1.5×10^{6} (5.5×10^{4})	3.1×10^{6} (1.6×10^{5})	2.3×10^{6} (1.2×10^{5})	5.5×10^{6} (3.9×10^{5})	1.7×10^{7} (1.3×10^{6})

Supplementary Table 2B: Abundances of bacterial 16S rRNA genes per liter in the 0.2 µm and 0.1 µm filter fraction of seepage samples taken between November 2016 and March 2017. Data are means (± s. d.) of three technical replicates. n. a.: no samples available/samples were not analyzed for bacterial 16S rRNA gene abundances.

lysimeter	filter fraction	11/16/2016	11/30/2016	12/28/2016	01/25/2017	02/08/2017	02/20/2017	03/01/2017	03/08/2017
**H1L1-2**	0.2 µm	1.1×10^{10} (7.9×10^{8})	1.1×10^{10} (2.2×10^{8})	3.5×10^{9} (9.3×10^{7})	6.9×10^{9} (5.7×10^{8})	5.4×10^{9} (2.1×10^{7})	1.5×10^{10} (5.9×10^{8})	n. a.	n. a.
	0.1 µm	1.2×10^{8} (1.8×10^{6})	6.2×10^{7} (4.5×10^{4})	5.8×10^{7} (3.6×10^{6})	3.2×10^{7} (1.7×10^{6})	3.2×10^{7} (9.0×10^{5})	5.7×10^{7} (3.9×10^{6})	n. a.	n. a.
**H1L3-1**	0.2 µm	n. a.	n. a.	n. a.	n. a.	n. a.	n. a.	4.0×10^{9} (1.7×10^{8})	4.4×10^{9} (2.4×10^{8})
	0.1 µm	n. a.	n. a.	n. a.	n. a.	n. a.	n. a.	4.5×10^{6} (4.4×10^{5})	2.6×10^{7} (2.6×10^{6})
**H1L3-2**	0.2 µm	n. a.	n. a.	n. a.	n. a.	n. a.	n. a.	4.2×10^{9} (2.4×10^{8})	5.2×10^{9} (3.2×10^{8})
	0.1 µm	n. a.	n. a.	n. a.	n. a.	n. a.	n. a.	3.6×10^{6} (8.9×10^{5})	2.4×10^{7} (1.3×10^{6})
**H2L1-1**	0.2 µm	n. a.	n. a.	n. a.	n. a.	6.4×10^{9} (8.1×10^{8})	n. a.	n. a.	n. a.
	0.1 µm	n. a.	n. a.	n. a.	n. a.	6.2×10^{6} (3.7×10^{6})	n. a.	n. a.	n. a.
**H2L1-2**	0.2 µm	n. a.	n. a.	n. a.	n. a.	n. a.	n. a.	9.9×10^{9} (7.9×10^{8})	n. a.
	0.1 µm	n. a.	n. a.	n. a.	n. a.	n. a.	n. a.	1.5×10^{7} (8.9×10^{5})	n. a.
